# Effects of vitamin D levels and vitamin D supplementation on allergic diseases: an umbrella review

**DOI:** 10.3389/falgy.2026.1841244

**Published:** 2026-06-29

**Authors:** Yuxin Hou, Qitong Zhang, Xianpeng Xu, Guangran Zhao

**Affiliations:** 1 The First Affiliated Hospital of Heilongjiang University of Chinese Medicine, Harbin, China; 2 The Second Affiliated Hospital of Heilongjiang University of Chinese Medicine, Harbin, China; 3 The Quzhou Affiliated Hospital of Wenzhou Medical University, Quzhou, China

**Keywords:** allergic diseases, allergic rhinitis, umbrella review, vitamin D, vitamin D supplementation

## Abstract

**Background:**

The association between vitamin D levels/supplementation and the risk of allergic diseases has been extensively studied. However, the strength and consistency of the existing evidence remain unclear, and most reviews have focused on single diseases. This umbrella review aims to systematically evaluate the current meta-analytic evidence on the associations between vitamin D and allergic diseases, in order to establish a hierarchy of evidence and identify research gaps.

**Methods:**

PubMed, Embase, Cochrane Library, and Web of Science were systematically searched from inception to October 21, 2025. Summary effect estimates, 95% confidence intervals, I^2^ statistic, 95% prediction intervals, small study effects, *p*-value of the largest study, and excess significance bias were recalculated. The methodological quality of included studies was appraised using AMSTAR 2 (A Measurement Tool to Assess Systematic Reviews 2).

**Results:**

A total of 14 eligible articles were included, yielding 16 associations (total population: 66,387). The credibility of evidence assessment revealed the following: the association between serum 25(OH)D levels and urticaria was graded as highly suggestive evidence (MD −9.35, −12.27 to −6.44, class II); the associations between serum 25(OH)D levels and allergic rhinitis and between vitamin D supplementation and urticaria were graded as suggestive evidence (SMD −1.29, −1.92 to −0.65, class III). Vitamin D supplementation significantly alleviated symptoms in allergic rhinitis (SMD −2.25, −3.05 to −1.43, class Ⅳ), reduced disease severity in atopic dermatitis (SMD −0.41, −0.65 to −0.17, class Ⅳ), and decreased clinical scores in urticaria (SMD −3.63, −5.72 to −1.54, class III). However, no significant effect was observed for vitamin D supplementation on asthma or food allergy. Prenatal vitamin D supplementation showed no significant preventive effect on the risk of offspring allergic rhinitis, atopic dermatitis, or asthma.

**Conclusions:**

Lower vitamin D levels are consistently associated with several allergic diseases. The available evidence suggests that vitamin D supplementation demonstrates beneficial effects on symptom improvement for specific diseases (allergic rhinitis, atopic dermatitis, urticaria), but its efficacy is disease-specific. Future research requires greater standardization and precision to clarify the exact role and clinical utility of vitamin D in the prevention and management of different allergic diseases.

**Systematic Review Registration:**

https://www.crd.york.ac.uk/prospero/

## 1 Introduction

Vitamin D is an essential fat-soluble vitamin with a variety of biological functions (1), which not only regulates calcium and phosphorus metabolism and promotes the development of bones and teeth (2), but also plays an important role in immune regulation, prevention of cardiovascular diseases, metabolic diseases and tumor treatment (3–5). In addition to light and food, vitamin D is also available through artificial supplements (including oral, injectable, and topical agents), which are taken in specific situations, such as areas with lack of sunlight or health problems that prevent the absorption of sufficient amounts of vitamin D (6).

Allergic diseases are a large group of diseases mainly mediated by immunoglobulin E (IgE) produced by atopic individuals in response to allergens, including allergic rhinitis (AR), asthma, atopic dermatitis (AD), and food allergy (7). Notably, all together, these now affect about 20% of the global population (8). Therefore, allergic diseases are both medical and public health problems. Allergic disease is a multifactorial disease with complex pathophysiology and is the result of a combination of genetic factors, immune responses and multiple environmental factors (9). The mechanism of vitamin D in allergy and immunity has been established (10, 11). Actually, it has been proposed that vitamin D insufficiency contributes to the increase in asthma and allergic diseases (12), and various biological mechanisms have been proposed for the role of vitamin D in the development and treatment of asthma and allergies (13).

Based on the relationship between vitamin D and allergic diseases and the effect of vitamin D on allergic diseases, many studies have been meta-analyzed to investigate the relationship between vitamin D levels and allergic diseases and the effect of vitamin D supplementation on allergic diseases (14–16). Although these studies have been summarized by meta-analyses, most of these meta-analyses focused on specific allergic diseases and did not scrutinize publication bias or reporting bias. To date, there has been a lack of standardized and definitive studies on the quality of evidence on vitamin D and allergic diseases. Therefore, to provide a comprehensive body of evidence for clinicians, researchers, and policymakers, a systematic identification of relevant meta-analyses, synthesis of their findings, and appraisal of the consistency and certainty of the evidence were performed through a comprehensive review of the meta-analyses. Accordingly, this umbrella review offers several novelties and strengths. First, to the best of the authors’ knowledge, this is the first umbrella review to comprehensively synthesize the associations between vitamin D (both serum levels and supplementation) and a wide spectrum of allergic diseases, including allergic rhinitis, asthma, atopic dermatitis, urticaria, food allergy, and others. Second, unlike previous narrative summaries, a predefined grading system was applied to classify the credibility of the evidence (from convincing to not significant), providing a transparent hierarchy of the existing findings. Third, observational and interventional evidence were concurrently evaluated, allowing for a more integrated understanding of both association and potential intervention effects. The primary aim of this study is to systematically evaluate the current meta-analytic evidence on the associations between vitamin D and allergic diseases, in order to establish a hierarchy of evidence and identify research gaps.

## 2 Methods

This umbrella review followed the PRIOR (Preferred Reporting Items for Overviews of Reviews) guidelines (appendix pp 1–2) (17). This review has been registered on the PROSPERO (CRD42024567148). The screening process, data extraction and methodological evaluation of the articles were conducted independently by two researchers (YXH and TQZ) and any disagreements were resolved through discussions with a third researcher (GRZ).

### 2.1 Search strategy and eligibility criteria

PubMed, Embase, Cochrane Library, and Web of Science databases were systematically searched from inception to October 21, 2025. The references of eligible articles and relevant gray literature were also manually searched. The aim was to investigate meta-analyses examining the effect of vitamin D on outcomes of various allergic diseases. Therefore, search keywords such as “vitamin D”, “allergic disease”, “meta-analysis” and “systematic review” were used; the full search strategy for each database is presented in the appendix (p 3).

Only systematic reviews or meta-analyses that reported the effects of vitamin D levels and vitamin D supplementation on allergic diseases were included. There were no restrictions on any language. Randomized controlled trials (RCTs), cohort studies, case-control studies, and cross-sectional studies were included. Moreover, to more comprehensively appraise the association between vitamin D and allergic disease, studies that investigated vitamin D status during pregnancy and the risk of allergic disease in offspring, as well as the effects of vitamin D supplementation during pregnancy on the outcome of allergic disease in offspring, were also included. The allergic diseases selected were those that are more common clinically, which included asthma, wheezing, allergic rhinitis, atopic dermatitis, eczema, food allergy, and urticaria. Articles that did not investigate the effects of vitamin D levels and supplementation on allergic disease were excluded. Non-human studies, conference abstracts, letters, study protocols, and primary studies were also excluded. Furthermore, articles that did not have a meta-analysis or did not present sufficient data were excluded.

When two or more reviews study an identical topic, it is difficult to avoid including the same primary study multiple times, which may lead to overlapping of reviews. Inclusion of reviews with overlapping associations may result in biased results. Quantitative studies that conducted meta-analyses were prioritized over qualitative studies. Additionally, more than half of the published reviews were out of date after 5.5 years (18). Therefore, for two or more reviews on the same topic, outdated reviews (reviews published before 2019) were excluded. For reviews on the same topic that were not outdated, the degree of overlap was assessed using the corrected covered area (CCA) (19). The formula is CCA=(n-r)/(rc-r), where *n* is the number of primary studies included in the reviews, r is the number of primary studies after removal of duplicates (number of rows), and c is the number of reviews (number of column). The CCA was calculated to be in the range of 0% to 5% for slight overlap, 6% to 10% for moderate overlap, 11% to 15% for high overlap, and >15% for very high overlap. If there was a high degree of overlap between two or more reviews, the review with the higher AMSTAR 2 (A Measurement Tool for Assessing Systematic Reviews 2) (20) score was chosen; if the scores were the same, the one that contained more studies was selected. For associations with moderate overlap (CCA 6%–10%), all relevant reviews were to be retained and their results compared qualitatively to assess the robustness of the conclusions, because excluding any single review might lead to loss of unique primary studies. No formal quantitative sensitivity analysis was performed; instead, the consistency of findings across overlapping reviews would serve as a sensitivity assessment.

### 2.2 Data extraction

Two investigators (YXH and TQZ) independently extracted the data from the eligible articles. Data extracted included: first author, year of publication, sample size, number of participants, number and type of studies included in the study, assessment tools used, methods of analysis, results of assessment, heterogeneity, and outcomes. Cross-checks were conducted, and any discrepancies were resolved through discussion with a third investigator (GRZ).

### 2.3 Data analysis

For eligible meta-analyses for which data were available, effect sizes for the individual studies reported in each meta-analysis were obtained, and the summary effect sizes were reanalyzed using a random-effects model (DerSimonian-Laird method). The 95% confidence intervals (CIs) and *p*-values were also calculated. The I^2^ statistic was calculated using the Cochrane Q test for heterogeneity between studies (I^2^ > 50% indicates high heterogeneity) (21). The 95% prediction intervals were estimated, which further explain the heterogeneity between studies and assess the expected uncertainty in effect estimates for studies with the same association (22, 23). The small study effect was assessed (*p* > 0.10 indicates no small study effects), which refers to the fact that studies with small sample sizes have larger effect sizes than studies with larger sample sizes (24). Excess significance bias was calculated, which is a measure of literature bias that compares the expected and observed number of statistically significant individual studies (*p* > 0.10 indicates no excessive significance bias) (25). All analyses of the data were conducted with R version 4.2.3 and its packages.

### 2.4 Quality assessment and the credibility of evidence

For eligible reviews, the methodological quality was assessed using AMSTAR 2 (20), which is a tool consisting of 16 items with excellent consistency, reliability, and validity. Of the 16 items, 7 of the items were key items, that is, items 2, 4, 7, 9, 11, 13, and 15. Each item was evaluated as “yes,” “no,” and “partially yes,” and its methodological quality was rated as high, moderate, low, and critically low on the basis of the evaluation of key and non-key items.

In accordance with previous umbrella reviews (26–29), the level of the credibility of evidence was classified based on the characteristics of randomized controlled studies and observational studies into five classes: convincing (class I), highly suggestive (class II), suggestive (class III), weak (class IV), and not significant (NS). Class I: number of cases > 1,000, random effects *p*-value < 0.000001, *p*-value of the largest study < 0.05, heterogeneity (I^2^) < 50%, prediction intervals excluding the null, no excessive significance bias, no small study effects. Class II: number of cases > 1,000, random effects *p*-value < 0.000001, *p*-value of the largest study < 0.05. Class III: number of cases > 1,000, random effects *p*-value < 0.001. Class IV: random effects *p*-value < 0.05. NS (not |significant): random effects *p*-value > 0.05.

## 3 Results

### 3.1 Search results

**Figure 1:**
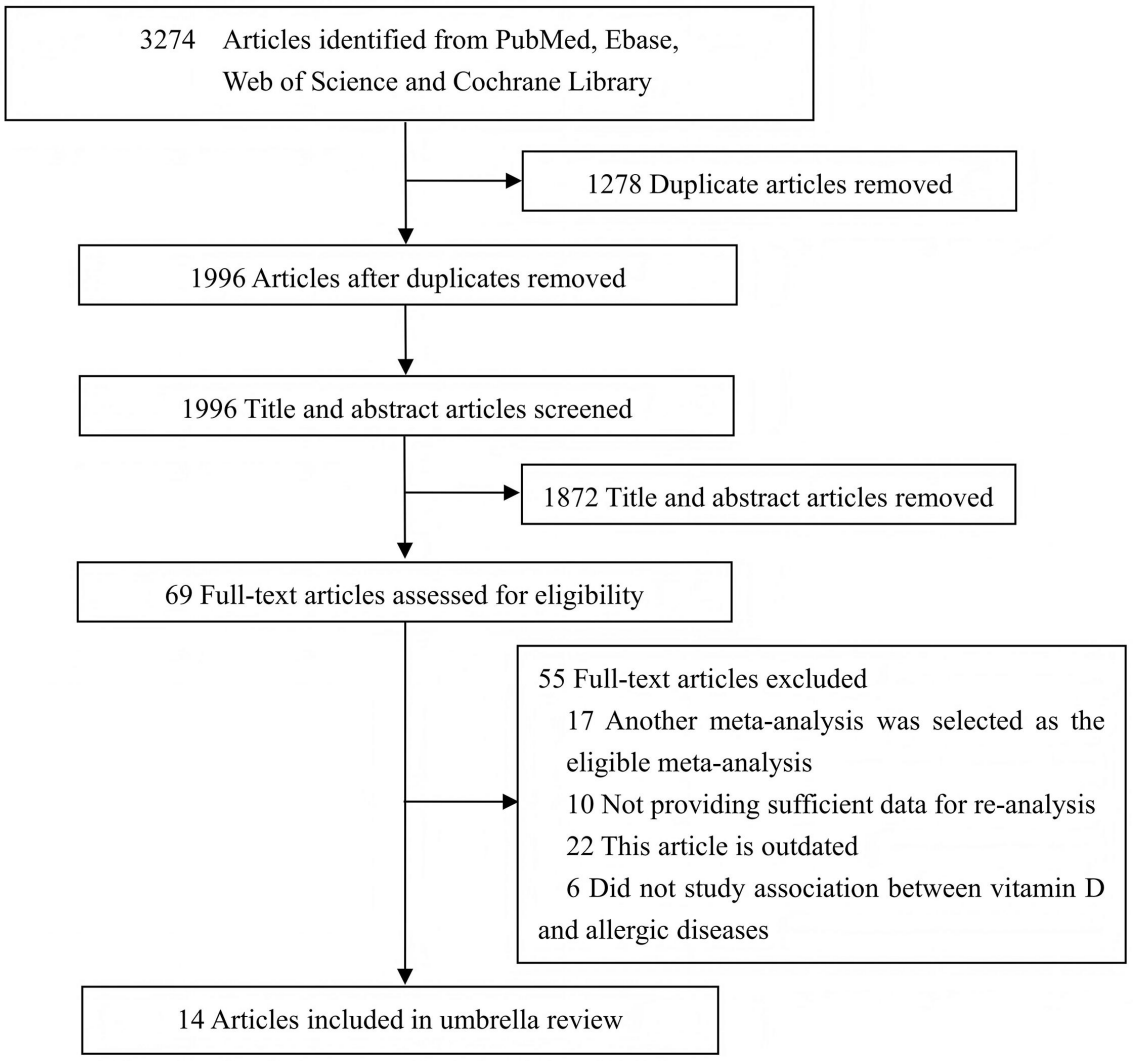
Flow diagram of search strategy and studies selection.

A total of 3,274 articles were retrieved. After removing 1,278 duplicate articles, as well as title and abstract screening, 88 articles were eligible for full-text screening,and finally 14 eligible articles were included in the review (Figure 1). The list of excluded articles by full-text screening with exclusion reasons and the list of excluded overlapping articles are provided in the appendix (pp 4–6).

### 3.2 Overlapping and non-overlapping associations

Review articles on the same topic were screened based on CCA, and 7 overlapping associations were identified (appendix pp 7–14). Overlapping associations included: vitamin D supplementation in allergic rhinitis (corrected covered area 11.76%, Supplementary Table S1), prenatal vitamin D supplementation and offspring allergic rhinitis (corrected covered area 100%, Supplementary Table S2), vitamin D supplementation in atopic dermatitis (corrected covered area 19.17%, Supplementary Table S3), serum 25 (OH) D levels and atopic dermatitis (corrected covered area 40.00%, Supplementary Table S4), prenatal vitamin D supplementation and offspring atopic dermatitis (corrected covered area 100%, Supplementary Table S5), vitamin D supplementation and asthma (corrected covered area 12.70%, Supplementary Table S5), and prenatal vitamin D supplementation and offspring asthma (corrected covered area 19.05%, Supplementary Table S5).

Study characteristics of reviews and statistical test results.

In total, 14 articles (30–43) were included in this umbrella review. These 14 articles yielded a total of 16 associations. These analyses collectively assessed the associations between two main exposure factors [serum 25(OH)D levels and vitamin D supplementation] and allergic diseases outcomes. All included meta-analyses were evaluated using AMSTAR 2. The study populations were primarily from multiple regions including Asia, Europe, and North America. The study designs of the included meta-analyses encompassed observational studies (such as case-control studies, cohort studies, cross-sectional studies) and interventional studies (randomized controlled trials). The general characteristics of each meta-analysis are detailed in Tables 1, 2.

**Table 1:** General characteristics of meta-analyses evaluating the association between serum 25 (OH) D levels and allergic diseases.

Author, year	Countries or regions	Study design	Objective	Exposure	Outcomes	Quality appraisal tool	Results summary	AMSTAR 2
Ju 2023 (30)	Pakistan, Iran, India, Turkey, Korea, China	Case-control	To investigate the role of vitamin D levels and the development of AR.	Serum 25 (OH) D	Allergic rhinitis	NOS	Lower vitamin D levels are associated with AR.	Low
Pacheco-González 2018 (31)	America, United Kingdom, France, Denmark, Taiwan,	Cohort	To investigate pregnancy serum vitamin D levels and the risk of allergic rhinitis in the offspring.	Pregnancy serum 25 (OH) D	Offspring allergic rhinitis	NOS	There is no association between prenatal vitamin D levels and the risk of developing allergic rhinitis in childhood.	Critically low
Chun Ng 2022 (32)	Spain, Egypt, China Germany, Turkey, India, Italy, Iran, Hong Kong, Korea, Egypt, Multi	Cohort, case-control, cross-sectional	To investigate the association between serum 25(OH)D level and AD.	Serum 25 (OH) D	Atopic dermatitis	NOS	It was found that 25(OH)D levels were significantly lower in AD patients compared to healthy controls.	High
Hidajat 2024 (33)	Australia, France, Germany Denmark, Taiwan, Poland Australia, America	Cohort	To evaluate the association between maternal cord blood 25(OH)D levels and the risk of AD.	Maternal cord blood 25(OH)D	Offspring atopic dermatitis	STROBE	Maternal cord blood 25(OH)D levels < 50 nmol/L were associated with a greater risk of developing AD.	Low
Wang 2021 (34)	Denmark, Turkey, China, Mongolian, German, Nigeria, America, Ireland, Poland, Korea, Iran, Canada, India, Italy	Case-control	To investigate the association between serum 25(OH)D level and asthma.	Serum 25 (OH) D	Asthma	NOS	Children with asthma have significantly lower 25(OH)D levels than non-asthmatic children.	Low
Li 2021 (35)	America, India, Poland, Egypt, Thailand, United Kingdom, Turkey, Korea, Iran, Turkey, China	Case-control	To investigate the association between serum 25(OH)D level and urticaria.	Serum 25 (OH) D	Urticaria	NOS	The urticaria population may be associated with a high risk of lower serum 25(OH)D.	Moderate
Daneshvar 2024 (36)	Ltaly, Turkey, India, Iran, Congo	Case-control, cross-sectional	To investigate the association between serum 25(OH)D level and VKC.	Serum 25 (OH) D	Vernal keratoconjunctivitis	JBI	Serum vitamin D levels were lower in VKC patients compared to healthy controls.	High
Zheng 2025 (37)	Europe, South America, Asia,	Case-control, cross-sectional	To investigate the association between serum 25(OH)D level and cow's milk protein allergy	Serum 25 (OH) D	Cow's milk protein allergy	NOS	The vitamin D level was lower in the CMPA group than in the control group	Low

25 (OH) D, 25-HydroxyvitaminD; NOS, Newcastle-Ottawa Scale; STROBE, Strengthening the Reporting of Observational Studies in Epidemiology; AD, atopic dermatitis; AR, allergic rhinitis. VKC, vernal keratoconjunctivitis; CMPA, cow's milk protein allergy.

**Table 2:** General characteristics of meta-analyses evaluating the association between vitamin D supplementation and allergic diseases.

Author, year	Countries or regions	Study design	Comparator (vitamin D supplementation or level)	intervention	Outcome	Quality appraisal tool	Results summary	AMSTAR 2
Luo 2022 (38)	Denmark, United Kingdom, America	RCT	Supplement vs. placebo or nothing	Vitamin D supplementation in pregnant women	Infants allergic rhinitis	NA	Vitamin D supplementation in pregnant women has no effect on the development of allergic rhinitis prevention in infants.	Low
Surayya 2025 (39)	America, India, Indonesia, Mexico, Iran, Egypt, China	RCT, cohort	Adjuvant vitamin D vs. standard therapy	Adjuvant vitamin D supplementation	Allergic rhinitis	NOS	Vitamin D adjuvant therapy significantly alleviated nasal symptoms	Critically low
Nielsen 2024 (40)	Britain, Iran, Mongolia, Italy, Mexico, Canada, Egypt, India, Chile	RCT	High dose vs. low dose	Vitamin D supplementation	Atopic dermatitis	RoB	Vitamin D supplementation significantly reduced the severity of AD compared to controls.	High
Tareke 2020 (41)	Denmark, United Kingdom, America	RCT	High dose vs. low dose or placebo	Vitamin D supplementation in pregnant women	Infants atopic dermatitis	RoB	Vitamin D supplementation in pregnant women has no effect on the development of atopic dermatitis prevention in infants.	Low
Wang 2023 (42)	Iceland, Finland,	RCT, cohort	High dose vs. low dose	Vitamin D supplementation	Food allergy	RoB	There was no association between high-dose vitamin D supplementation and the risk of food allergy at 12 months.	Moderate
Williamson 2023 (43)	Canada, Spain, New Zealand, India, Poland, China, Mexico, Ireland, Japan,	RCT	Vitamin D versus placebo	Vitamin D supplementation	Asthma	RoB	Administration of vitamin D did not reduce the proportion of participants with asthma attacks	High
Tareke 2020 (41)	Denmark, United Kingdom, America	RCT	High dose vs. low dose or placebo	Vitamin D supplementation in pregnant women	Infants asthma	RoB	Vitamin D intake during pregnancy is not significantly associated with the development of asthma in offspring .	Low
Li 2021 (35)	America, India, Egypt	RCT	High dose vs. low dose or placebo	Vitamin D supplementation	Urticaria	Jadad	There was a significant decrease in clinical urticaria scores after the vitamin D intervention.	Moderate

RCT, randomized controlled trial; NA, not available; RoB Cochrane risk-of-bias tool for randomized trials. NOS, Newcastle-Ottawa Scale.

The 16 meta-analyses examining the associations between vitamin D (serum levels or supplementation) and allergic diseases were based on 66,387 total population. The number of primary studies included in each meta-analysis ranged from 3 to 24. The effect metrics were standardized mean difference (SMD), weighted mean difference (WMD), mean difference (MD), odds ratio (OR), and risk ratio (RR). Of the 16 meta-analyses, 10 (63%) reported statistically significant summary associations under the random effects model (*p* < 0.05). Ten (63%) of 16 associations showed large heterogeneity (I^2^ > 50%). Tests for small-study effects (Egger's test) and excess significance bias (ESB) were reported for most associations; their results were mixed, indicating the presence of these biases in some but not all analyses. The detailed statistical test results for each specific association between vitamin D exposure and allergic diseases are presented in Table 3.

**Table 3:** Summary findings for each systematic review with meta-analysis, with details of statistical test results.

Exposure	Author, year	Number of cases (intervention)/ total population	Number of study	Effect metrics	Random effects summary estimate (95% CI)	Random effects *p* value	*I^2^*	95% prediction interval	Egger *p*-value	ESB *p-*value	*p*-value of the largest study	Whether the 95% PI includes the null value	Class of evidence
Serum 25 (OH) D level and allergic diseases													
Allergic rhinitis	Ju 2023 (30)	1504/2,939	12	SMD	−1.29 (−1.92 to −0.65)	7.09e-05	98%	(−3.98 to 1.41)	5.43e-02	1.44e-02	<0.05	Null	Class III
Atopic dermatitis	Ng 2022 (32)	1,478/2,468	14	WMD	−7.42 (−11.91 to −2.92)	1.22e-03	99%	(−26.49 to 11.66)	6.69e-01	4.72e-01	<0.05	Null	Class Ⅳ
Asthma	Wang 2021 (34)	5,711/27,272	24	SMD	−1.36 (−2.38 to −0.35)	8.29e-03	99%	(−6.70 to 3.98)	7.56e-01	9.41e-02	<0.05	Null	Class Ⅳ
Urticaria	Li 2021 (35)	1,945/7,539	17	MD	−9.35 (−12.27 to −6.44)	1.45e-09	99%	(−22.19 to 3.48)	8.80e-01	2.18e-01	<0.05	Null	Class Ⅱ
Vernal keratoconjunctivitis	Daneshvar 2024 (36)	266/568	6	Cohen's d	−0.92 (−1.16 to −0.69)	5.26e-15	41%	(−1.53 to −0.32)	5.38e-01	2.80e-01	<0.05	Not null	Class Ⅳ
Cow's milk protein allergy	Zheng 2025 (37)	605/1,163	12	SMD	−1.22 (−1.98 to −0.45)	1.91e-03	97%	(−4.29 to 1.86)	1.49e-01	2.13e-02	<0.05	Null	Class Ⅳ
Prenatal vitamin D level and offspring allergic diseases													
Allergic rhinitis	Pacheco 2018 (31)	NR/7,978	6	OR	1.02 (0.82 to 1.26)	8.77e-01	17%	(0.62 to 1.67)	6.33e-02	6.96e-01	>0.05	Null	NS
Atopic dermatitis	Hidajat 2024 (33)	NR/3,952	5	OR	1.60 (1.15 to 2.22)	4.82e-03	0%	(0.94 to 2.72)	4.66e-01	5.79e-01	>0.05	Null	Class Ⅳ
Vitamin D supplementation													
Allergic rhinitis	Surayya 2025 (39)	442/893	14	SMD	−2.25 (−3.05 to −1.43)	5.83e-08	96%	(−5.55 to −1.06)	5.54e-03	1.16e-05	<0.05	Null	Class Ⅳ
Atopic dermatitis	Nielsen 2024 (40)	347/684	11	SMD	−0.41 (−0.65 to 0.17)	9.94e-04	58%	(−1.16 to 0.34)	1.99e-01	6.82e-01	<0.05	Null	Class Ⅳ
Food allergy	Wang 2023 (42)	NR/2,423	3	OR	0.84 (0.71 to 1.01)	6.05e-02	0%	(0.27 to 2.68)	4.23e-01	2.27e-01	>0.05	Null	NS
Asthma	Williamson 2023 (43)	888/1,778	14	OR	1.04 (0.81 to 1.34)	7.54e-01	0%	(0.79 to 1.39)	4.17e-01	7.43e-01	>0.05	Null	NS
Urticaria	Li 2021 (35)	115/230	3	SMD	−3.63 (−5.72 to −1.54)	6.81e-04	96%	(−29.51 to 22.26)	4.74e-02	2.07e-03	<0.05	Null	Class III
Prenatal vitamin D supplementation and offspring allergic diseases													
Allergic rhinitis	Luo 2022 (38)	689/1,402	3	RR	0.99 (0.68 to 1.46)	9.73e-01	47%	(0.01 to 50.62)	6.75e-01	7.84e-01	>0.05	Null	NS
Atopic dermatitis	Tareke 2020 (41)	1,111/2,200	4	OR	0.95 (0.82 to 1.10)	5.02e-01	0%	(0.69 to 1.31)	6.02e-01	6.79e-01	>0.05	Null	NS
Asthma	Tareke 2020 (41)	1,461/2,898	6	OR	0.89 (0.69 to 1.15)	3.71e-01	46%	(0.45 to 1.75)	7.63e-01	8.45e-01	>0.05	Null	NS

NR, not reported; OR, odds ratio; RR, risk ratio; SMD, standard mean difference; WMD, weighted mean difference; NS, not significant.

### 3.3 Association between serum 25(OH)D levels and allergic diseases

Meta-analyses consistently reported lower serum 25(OH)D levels in patients with allergic diseases compared to healthy controls (Table 3). For AR, the SMD was −1.29 (95% CI: −1.92 to −0.65; *p* < 0.05). For AD, the WMD was −7.42 (95% CI: −11.91 to −2.92; *p* < 0.05). For asthma, the SMD was −1.36 (95% CI: −2.38 to −0.35; *p* < 0.05). For urticaria, the MD was −9.35 (95% CI: −12.27 to −6.44; *p* < 0.05). For vernal keratoconjunctivitis (VKC), the Cohen's d was −0.92 (95% CI: −1.16 to −0.69; *p* < 0.05). For cow's milk protein allergy (CMPA), the SMD was −1.22 (95% CI: −1.98 to −0.45; *p* < 0.05).

Association between prenatal vitamin D levels and offspring allergic diseases.

No significant association was found between maternal serum 25(OH)D levels during pregnancy and the risk of allergic rhinitis in offspring (OR=1.02, 95% CI: 0.82 to 1.26; *p* > 0.05; NS). Maternal cord blood 25(OH)D levels were associated with a significantly increased risk of atopic dermatitis in offspring (OR=1.60, 95% CI: 1.15 to 2.22; *p* < 0.05).

### 3.4 Efficacy of vitamin D supplementation on allergic diseases

For AR, adjuvant vitamin D supplementation significantly alleviated nasal symptoms (SMD=−2.25, 95% CI: −3.05 to −1.43; *p* < 0.05). For AD, vitamin D supplementation significantly reduced disease severity (SMD=−0.41, 95% CI: −0.65 to −0.17; *p* < 0.05). For urticaria, vitamin D supplementation led to a significant decrease in clinical scores (SMD=−3.63, 95% CI: −5.72 to −1.54; *p* < 0.05). No significant protective or therapeutic effect was observed for vitamin D supplementation on asthma (OR=1.04, 95% CI: 0.81 to 1.34; *p* > 0.05) and food allergy (OR=0.84, 95% CI: 0.71 to 1.01; *p* > 0.05).

Efficacy of prenatal vitamin D supplementation and offspring allergic diseases.

No significant protective or therapeutic effect was observed for prenatal vitamin D supplementation on offspring AR (RR = 0.99, 95% CI: 0.68 to 1.46; *p* > 0.05; NS), AD (OR=0.95, 95% CI: 0.82 to 1.10); *p* > 0.05) and asthma (OR=0.89, 95% CI: 0.69 to 1.15; *p* > 0.05).

### 3.5 Quality assessment and credibility of evidence

All eligible articles were assessed using the AMSTAR 2 (appendix p 15). Of the 14 meta-analysis articles of vitamin D levels and vitamin D supplementation on allergic diseases (Supplementary Table S8), 5 (36%) were graded as high quality, 3 (21%) were graded as moderate quality, 4(29%) were graded as low quality, and 2 (14%) were graded as critically low quality.

**Figure 2:**
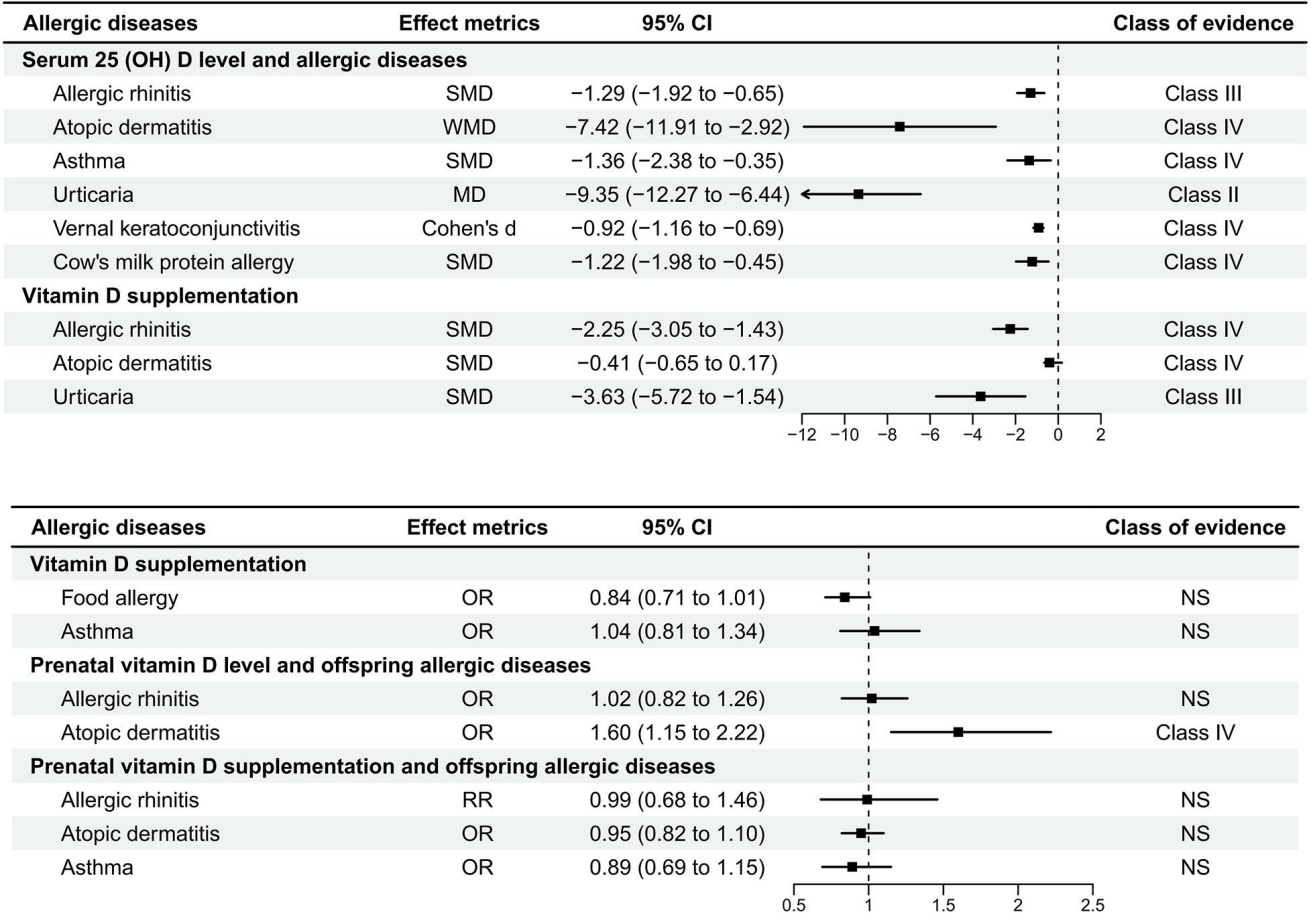
Summary effect sizes of vitamin D levels and vitamin D supplementation on allergic diseases.

Of the 16 associations, one association was graded as highly suggestive evidence (class II; Table 3): serum 25 (OH) D level and urticaria; two associations were graded as suggestive evidence (class III; Table 3, Figure 2): serum 25 (OH) D level and AR, and vitamin D supplementation and urticaria; seven associations were graded as weak evidence (classⅣ; Table 3, Figure 2): serum 25 (OH) D level and AD, serum 25 (OH) D level and asthma, serum 25 (OH) D level and VKC, serum 25 (OH) D level and CMPA, prenatal vitamin D level and offspring AD, vitamin D supplementation and AR, and vitamin D supplementation and AD; six associations were graded as not significant evidence (NS; Table 3, Figure 2): prenatal vitamin D level and AR, vitamin D supplementation and food allergy, vitamin D supplementation and asthma, prenatal vitamin D supplementation and AR, prenatal vitamin D supplementation and AD, and prenatal vitamin D supplementation and asthma.

## 4 Discussion

This umbrella review provides a comprehensive assessment of the current meta-analytic evidence on the associations between vitamin D [serum 25 (OH) D level and supplementation] and allergic diseases. Our synthesis reveals that lower serum vitamin D levels are consistently associated with the presence of several allergic conditions, including AR, AD, asthma, urticaria, VKC, and CMPA. Regarding interventions, vitamin D supplementation demonstrates beneficial effects on symptom alleviation in AR, AD, and urticaria, but shows no significant protective or therapeutic effect on asthma or food allergy. Furthermore, maternal vitamin D status or supplementation during pregnancy did not show consistent or significant protective associations with the risk of offspring allergic diseases (AR, AD, asthma). Collectively, these findings indicate that the role of vitamin D in the pathophysiology of allergic diseases is disease-specific and context-dependent, and its potential as a preventive or therapeutic target warrants cautious evaluation.

The consistent observation in this review is the generally lower serum 25(OH)D concentrations among patients with allergic diseases. This inverse association reached statistical significance for AR, AD, asthma, urticaria, VKC, and CMPA. This pattern is consistent with the hypothesis that vitamin D insufficiency may play a role in the development or exacerbation of allergic disorders (44). However, these observational associations do not inherently imply causation, as they may be influenced by residual confounding, reverse causation, or other unmeasured factors. The active form of vitamin D, 1,25-dihydroxyvitamin D3, exerts immunomodulatory effects by binding to the vitamin D receptor (VDR), which is widely expressed on various immune cells including dendritic cells, T cells, and B cells (45–47). The vitamin D–VDR signaling pathway promotes the differentiation and function of regulatory T cells (Tregs), while suppressing the overactivation of T helper 2 (Th2) cells and the production of related cytokines such as IL-4, IL-5, and IL-13 (48, 49). Furthermore, this pathway contributes to the enhancement of epithelial barrier function (50). These mechanisms collectively highlight the crucial role of vitamin D in maintaining immune tolerance and restraining allergic inflammation (50–53). Consequently, insufficient levels of vitamin D may lead to dysregulated immune responses, increased susceptibility to allergens, and greater severity of allergic diseases. However, association does not equate to causation. The observed low vitamin D levels in observational studies could also be a consequence of chronic inflammation, behavioural changes due to increased disease activity, or be linked to other comorbid factors (54). Nevertheless, the beneficial effects of supplementation in some diseases lend some support to a potential causal relationship.

Regarding interventional efficacy, this review found positive effects of vitamin D supplementation on AR, AD, and urticaria. For instance, adjuvant vitamin D supplementation significantly alleviated nasal symptoms in AR. In patients with AD, supplementation was associated with a significant reduction in disease severity. For chronic urticaria, vitamin D supplementation also showed a substantial decrease in clinical scores. These results suggest that correcting vitamin D insufficiency may directly modulate the immunopathological processes in these diseases, particularly as an adjunctive therapy in already-established disease. Conversely, vitamin D supplementation showed no significant benefit for asthma or food allergy. This “disease-specific” effect may stem from multiple factors. First, the pathogenesis of asthma and food allergy may involve more complex or earlier (e.g., early-life immune programming) pathological processes that are not easily reversed by later-life vitamin D supplementation alone (55, 56). Second, high heterogeneity exists across studies in terms of intervention timing, dosage, duration, baseline vitamin D status, and patient populations (e.g., age, asthma phenotype, allergen types), which may obscure effects in specific subgroups (57). Third, the clinical endpoints in trials for these diseases (e.g., asthma exacerbation rate, confirmed food allergy) might be less sensitive to vitamin D intervention (58). Notably, the evidence for the association between vitamin D supplementation and asthma in this review was graded as “not significant” with low heterogeneity, strengthening the conclusion that supplementation offers no universal therapeutic benefit for established asthma.

This review did not find stable, significant protective associations between maternal serum vitamin D levels or supplementation during pregnancy and the risk of offspring AR, AD, or asthma. Only one analysis suggested an association between low cord blood vitamin D and increased risk of offspring AD. This overall null finding is not entirely aligned with the expectations of the “developmental origins hypothesis,” which posits that early life (including the fetal period) is a critical window for immune system development and programming, where maternal nutritional status can have long-lasting effects on offspring allergy risk (59, 60, 61).

The lack of evidence may stem from: 1) residual confounding in observational studies (e.g., genetic background, maternal diet, socioeconomic status, other environmental exposures) that is difficult to fully control; 2) substantial variations in the timing (trimester), dose, and frequency of supplementation in intervention studies; 3) inconsistent ages at outcome assessment and diagnostic criteria for offspring; 4) potential effect modification by specific genetic (e.g., VDR polymorphisms) or environmental (e.g., latitude, sunlight patterns) factors, diluting overall effects. Therefore, a potential influence of prenatal vitamin D on offspring allergy cannot be completely ruled out. Future research needs greater refinement to explore the effects of specific supplementation regimens (e.g., initiation at specific gestational weeks, specific doses) in specific high-risk populations.

## 5 Limitations

This review assessed the methodological quality (AMSTAR 2) and graded the credibility of evidence for the included meta-analyses. The results showed that only 36% of the meta-analyses were rated as high quality, and over half of the evidence was graded as having “weak” (Class IV) or “non-significant” (NS) credibility. This reflects the limited overall strength and consistency of the existing evidence base in this field. High heterogeneity was a prominent issue in most association analyses. In this umbrella review, quantitative subgroup analyses could not be performed due to inconsistent reporting across the included meta-analyses. However, potential sources were qualitatively synthesized, which likely include: differences in study designs (varying levels of confounding in cross-sectional, case-control, and cohort studies); diversity in populations (age, ethnicity, geographic location, baseline vitamin D status); variations in vitamin D measurement methods; lack of uniformity in diagnostic criteria for allergic diseases; and differences in statistical models. Although random-effects models and prediction intervals were used to synthesize and interpret this heterogeneity, it substantially reduces the precision of summary effect estimates and the credibility of the evidence. Furthermore, the limitations of this umbrella review are inherent to the limitations of the included primary meta-analyses, including potential publication bias, small-study effects, and residual confounding in the original studies. Although testing for some biases was performed, the results were mixed. For example, small-study effects or excess significance bias were detected in the analyses of vitamin D supplementation for AR and urticaria, suggesting the possibility of unpublished small negative results or selective reporting. It is important to note that while these tests indicate the presence of bias, they do not quantify the extent to which the summary effect estimates are distorted. Therefore, the findings from these associations, and consequently any clinical conclusions drawn from them, should be interpreted with particular caution.

## 6 Recommendations for future research

Based on the findings and limitations of this umbrella review, several priorities for future research are proposed. First, standardization and precision are urgently needed; future studies should adopt uniform assays for serum 25(OH)D measurement, consistent diagnostic criteria for allergic diseases, and well-defined protocols for vitamin D supplementation that specify timing, dosage, duration, and baseline vitamin D status. Second, mechanism-driven investigations are warranted to elucidate why vitamin D supplementation exerts beneficial effects in allergic rhinitis, atopic dermatitis, and urticaria but not in asthma and food allergy. Such knowledge could inform more targeted therapeutic strategies. Third, high-quality primary studies, particularly large, well-designed randomized controlled trials with long-term follow-up, are needed to address evidence gaps regarding prenatal vitamin D supplementation for offspring allergy prevention and vitamin D for food allergy, where current evidence remains weak or non-significant. Fourth, individualized approaches should be explored to identify patient subgroups defined by specific VDR genotypes, severe vitamin D deficiency, or particular disease phenotypes that may derive greater benefit from vitamin D supplementation. Addressing these research priorities will help definitively establish the clinical utility of vitamin D in the prevention and management of different allergic diseases.

## 7 Conclusion

This umbrella review systematically synthesizes the highest level of current evidence on the associations between vitamin D and allergic diseases. The findings indicate that low vitamin D status is consistently associated with several allergic conditions, and supplementation shows beneficial effects for symptom improvement in some diseases (allergic rhinitis, atopic dermatitis, urticaria) but limited effects in others (asthma, food allergy). Prenatal supplementation did not demonstrate clear preventive effects on offspring allergy. The strength of evidence for most associations was weak to moderate, marked by significant heterogeneity and potential biases. Future research should move towards greater standardization, precision, and mechanism-driven inquiry to definitively establish the exact role and clinical utility of vitamin D in the prevention and management of different allergic diseases, ultimately enabling more individualized and effective patient care strategies. The presence of these biases, although detected, could not be quantitatively corrected, which further underscores the need for caution when translating these findings into clinical practice.

## Data Availability

The original contributions presented in the study are included in the article/Supplementary Material, further inquiries can be directed to the corresponding author.
