# Supplementary material for: Effects of vitamin D levels and vitamin D supplementation on allergic diseases: an umbrella review
Source: Front Allergy. 2026 Jun 29;7:1841244. doi: 10.3389/falgy.2026.1841244 (PMC13357912; doi:10.3389/falgy.2026.1841244)

Supplementary appendix

Effects of vitamin D levels and vitamin D supplementation on allergic diseases: an umbrella review

1. PRIOR Checklist
2. Full search strategy
3. The list of excluded articles by full text screening with exclusion reason
4. The list of excluded overlapping and outdated articles
5. Table S1: Vitamin D supplementation in allergic rhinitis
6. Table S2: Prenatal vitamin D supplementation and offspring allergic rhinitis
7. Table S3: Vitamin D supplementation in atopic dermatitis
8. Table S4: Serum 25 (OH) D levels and atopic dermatitis
9. Table S5: Prenatal vitamin D supplementation and offspring atopic dermatitis
10. Table S6: Vitamin D supplementation and asthma
11. Table S7: Prenatal vitamin D supplementation and offspring asthma
12. Table S8: The results of the AMSTAR-2 assessment
13. Figure S1. Random-effects meta-analysis of the association between serum 25 (OH) D level and allergic rhinitis (forest plot and funnel plot)
14. Figure S2. Random-effects meta-analysis of the association between serum 25 (OH) D level and atopic dermatitis (forest plot and funnel plot)
15. Figure S3. Random-effects meta-analysis of the association between serum 25 (OH) D level and asthma (forest plot and funnel plot)
16. Figure S4. Random-effects meta-analysis of the association between serum 25 (OH) D level and urticaria (forest plot and funnel plot)
17. Figure S5. Random-effects meta-analysis of the association between serum 25 (OH) D level and vernal keratoconjunctivitis (forest plot and funnel plot)
18. Figure S6. Random-effects meta-analysis of the association between serum 25 (OH) D level and cow's milk protein allergy (forest plot and funnel plot)
19. Figure S7. Random-effects meta-analysis of the association between prenatal vitamin D level and offspring allergic rhinitis (forest plot and funnel plot)
20. Figure S8. Random-effects meta-analysis of the association between prenatal vitamin D level and offspring atopic dermatitis (forest plot and funnel plot)
21. Figure S9. Random-effects meta-analysis of the association between vitamin D supplementation and allergic rhinitis (forest plot and funnel plot)
22. Figure S10. Random-effects meta-analysis of the association between vitamin D supplementation and atopic dermatitis (forest plot and funnel plot)
23. Figure S11. Random-effects meta-analysis of the association between vitamin D supplementation and food allergy (forest plot and funnel plot)
24. Figure S12. Random-effects meta-analysis of the association between vitamin D supplementation and asthma (forest plot and funnel plot)
25. Figure S13. Random-effects meta-analysis of the association between vitamin D supplementation and urticaria (forest plot and funnel plot)
26. Figure S14. Random-effects meta-analysis of the association between prenatal vitamin D supplementation and allergic rhinitis (forest plot and funnel plot)
27. Figure S15. Random-effects meta-analysis of the association between prenatal vitamin D supplementation and atopic dermatitis (forest plot and funnel plot)
28. Figure S16. Random-effects meta-analysis of the association between prenatal vitamin D supplementation and asthma (forest plot and funnel plot)

PRIOR Checklist

(Gates M, Gates A, Pieper D, et al. Reporting guideline for overviews of reviews of healthcare interventions: development of the PRIOR statement. ***BMJ*** **2022;378:e070849. doi:10.1136/bmj-2022-070849.)**

**Section** Topic	#	**Item**	**Location reported**
**TITLE**			
Title	1	Identify the report as an overview of reviews.	Manuscript p 1
**ABSTRACT**			
Abstract	2	Provide a comprehensive and accurate summary of the purpose, methods, and results of the overview of reviews.	Manuscript p 1
**INTRODUCTION**			
Rationale	3	Describe the rationale for conducting the overview of reviews in the context of existing knowledge.	Manuscript p 2
Objectives	4	Provide an explicit statement of the objective(s) or question(s) addressed by the overview of reviews.	Manuscript p 3
**METHODS**			
Eligibility criteria	5a	Specify the inclusion and exclusion criteria for the overview of reviews. If supplemental primary studies were included, this should be stated, with a rationale.	Manuscript pp 2-3
	5b	Specify the definition of 'systematic review' as used in the inclusion criteria for the overview of reviews.	Manuscript pp 2-3
Information sources	6	Specify all databases, registers, websites, organizations, reference lists, and other sources searched or consulted to identify systematic reviews and supplemental primary studies (if included). Specify the date when each source was last searched or consulted.	Manuscript p 2
Search strategy	7	Present the full search strategies for all databases, registers and websites, such that they could be reproduced. Describe any search filters and limits applied.	Manuscript p 2<br>Appendix p 3
Selection process	8a	Describe the methods used to decide whether a systematic review or supplemental primary study (if included) met the inclusion criteria of the overview of reviews.	Manuscript pp 2-3
	8b	Describe how overlap in the populations, interventions, comparators, and/or outcomes of systematic reviews was identified and managed during study selection.	Manuscript p 3
Data collection process	9a	Describe the methods used to collect data from reports.	Manuscript p 3
	9b	If applicable, describe the methods used to identify and manage primary study overlap at the level of the comparison and outcome during data collection. For each outcome, specify the method used to illustrate and/or quantify the degree of primary study overlap across systematic reviews.	Manuscript p 3<br>Appendix pp 7-14
	9c	If applicable, specify the methods used to manage discrepant data across systematic reviews during data collection.	Manuscript p 3
Data items	10	List and define all variables and outcomes for which data were sought. Describe any assumptions made and/or measures taken to identify and clarify missing or unclear information.	Table 1<br>Table 2
Risk of bias assessment	11a	Describe the methods used to *assess* risk of bias or methodological quality of the included systematic reviews.	Manuscript p 3
	11b	Describe the methods used to *collect* data on (from the systematic reviews) and/or *assess* the risk of bias of the primary studies included in the systematic reviews. Provide a justification for instances where flawed, incomplete, or missing assessments are identified but not re-assessed.	Manuscript p 3
	11c	Describe the methods used to *assess* the risk of bias of supplemental primary studies (if included).	NA
Synthesis methods	12a	Describe the methods used to summarize or synthesize results and provide a rationale for the choice(s).	Manuscript p 3
	12b	Describe any methods used to explore possible causes of heterogeneity among results.	Manuscript p 6
	12c	Describe any sensitivity analyses conducted to assess the robustness of the synthesized results.	NA
Reporting bias assessment	13	Describe the methods used to *collect* data on (from the systematic reviews) and/or *assess* the risk of bias due to missing results in a summary or synthesis (arising from reporting biases at the levels of the systematic reviews, primary studies, and supplemental primary studies, if included).	Manuscript p 3
Certainty assessment	14	Describe the methods used to *collect* data on (from the systematic reviews) and/or *assess* certainty (or confidence) in the body of evidence for an outcome.	Manuscript p 3
**RESULTS**			
Systematic review and supplemental primary study selection	15a	Describe the results of the search and selection process, including the number of records screened, assessed for eligibility, and included in the overview of reviews, ideally with a flow diagram.	Manuscript p 3<br>Figure 1
	15b	Provide a list of studies that might appear to meet the inclusion criteria, but were excluded, with the main reason for exclusion.	Appendix pp 6 -8

**Section** Topic	**#**	**Item**	**Location reported**
Characteristics of systematic reviews and supplemental primary studies	16	Cite each included systematic review and supplemental primary study (if included) and present its characteristics.	Table 1 Table 2
Primary study overlap	17	Describe the extent of primary study overlap across the included systematic reviews.	Manuscript pp 3-4
Risk of bias in systematic reviews, primary studies, and supplemental primary studies	18a	Present assessments of risk of bias or methodological quality for each included systematic review.	Manuscript p 8
	18b	Present assessments (*collected* from systematic reviews or *assessed* anew) of the risk of bias of the primary studies included in the systematic reviews.	Manuscript p 8 Table 3
	18c	Present assessments of the risk of bias of supplemental primary studies (if included).	NA
Summary or synthesis of results	19a	For all outcomes, summarize the evidence from the systematic reviews and supplemental primary studies (if included). If meta-analyses were done, present for each the summary estimate and its precision and measures of statistical heterogeneity. If comparing groups, describe the direction of the effect.	Table 3 Figure 2
	19b	If meta-analyses were done, present results of all investigations of possible causes of heterogeneity.	Manuscript pp 8-9
	19c	If meta-analyses were done, present results of all sensitivity analyses conducted to assess the robustness of synthesized results.	NA
Reporting biases	20	Present assessments (*collected* from systematic reviews and/or *assessed* anew) of the risk of bias due to missing primary studies, analyses, or results in a summary or synthesis (arising from reporting biases at the levels of the systematic reviews, primary studies, and supplemental primary studies, if included) for each summary or synthesis assessed.	Manuscript pp 8-9
Certainty of evidence	21	Present assessments (*collected* or *assessed* anew) of certainty (or confidence) in the body of evidence for each outcome.	Manuscript p 9 Figure 2
**DISCUSSION**			
Discussion	22a	Summarize the main findings, including any discrepancies in findings across the included systematic reviews and supplemental primary studies (if included).	Manuscript pp 8-9
	22b	Provide a general interpretation of the results in the context of other evidence.	Manuscript p 9
	22c	Discuss any limitations of the evidence from systematic reviews, their primary studies, and supplemental primary studies (if included) included in the overview of reviews. Discuss any limitations of the overview of reviews methods used.	Manuscript p 9
	22d	Discuss implications for practice, policy, and future research (both systematic reviews and primary research). Consider the relevance of the findings to the end users of the overview of reviews, e.g., healthcare providers, policymakers, patients, among others.	Manuscript p 10
**OTHER INFORMATION**			
Registration and protocol	23a	Provide registration information for the overview of reviews, including register name and registration number, or state that the overview of reviews was not registered.	Manuscript p 2
	23b	Indicate where the overview of reviews protocol can be accessed, or state that a protocol was not prepared.	Manuscript p 2
	23c	Describe and explain any amendments to information provided at registration or in the protocol. Indicate the stage of the overview of reviews at which amendments were made.	NA
Support	24	Describe sources of financial or non-financial support for the overview of reviews, and the role of the funders or sponsors in the overview of reviews.	Manuscript p 11
Competing interests	25	Declare any competing interests of the overview of reviews' authors.	Manuscript p 11
Author information	26a	Provide contact information for the corresponding author.	Manuscriptp 1
	26b	Describe the contributions of individual authors and identify the guarantor of the overview of reviews.	Manuscript p 11
Availability of data and other materials	27	Report which of the following are available, where they can be found, and under which conditions they may be accessed: template data collection forms; data collected from included systematic reviews and supplemental primary studies; analytic code; any other materials used in the overview of reviews.	Appendix file

Full search strategy

PubMed/MEDLINE
((((((Vitamin D[MeSH Terms]) OR (Vitamin D[Title/Abstract])) OR (25-hydroxyvitamin D[Title/Abstract])) OR (25(OH)D[Title/Abstract])) OR (VD[Title/Abstract])) AND ((((((((((((((((((((allergic disease*[Title/Abstract]) OR (anaphylactic disease*[Title/Abstract])) OR (hypersensitivity disease*[Title/Abstract])) OR (atopic disease*[Title/Abstract])) OR (asthma[MeSH Terms])) OR (asthma*[Title/Abstract])) OR (wheeze[Title/Abstract])) OR (eczema[MeSH Terms])) OR (eczema[Title/Abstract])) OR (urticaria[MeSH Terms])) OR (urticaria[Title/Abstract])) OR (atopic dermatitis[Title/Abstract])) OR (allergic rhinitis*[Title/Abstract])) OR (allergic rhinoconjunctivitis[Title/Abstract])) OR (allergic conjunctivitis[Title/Abstract])) OR (allergic purpura[Title/Abstract])) OR (hay fever[Title/Abstract])) OR (allergy*[Title/Abstract])) OR (food allergy*[Title/Abstract])) OR (drug hypersensitivity[Title/Abstract])))) AND ((((((((((Meta-Analy*) OR (Meta-Analysis[Publication Type])) OR (Meta-Analysis as Topic[MeSH Terms])) OR (Meta-Analysis[Title/Abstract])) OR (Meta-Analyses[Title/Abstract])) OR (Systematic Review[Publication Type])) OR (Systematic Reviews as Topic[MeSH Terms])) OR (Systematic Review*[Title/Abstract]))))
Embase
('vitamin d'/exp OR 'vitamin d':ti,ab,kw OR '25-hydroxyvitamin d':ti,ab,kw OR ('25(OH)D':ti,ab,kw) OR vd:ti,ab,kw) AND ('allergic disease'/exp OR 'allergic disease*':ti,ab,kw OR 'anaphylactic disease*':ti,ab,kw OR 'hypersensitivity disease*':ti,ab,kw OR 'atopic disease*':ti,ab,kw OR asthma*:ti,ab,kw OR wheeze:ti,ab,kw OR eczema:ti,ab,kw OR urticaria:ti,ab,kw OR 'atopic dermatitis':ti,ab,kw OR 'allergic rhinitis*':ti,ab,kw OR 'allergic rhinoconjunctivitis':ti,ab,kw OR 'allergic conjunctivitis':ti,ab,kw OR 'allergic purpura':ti,ab,kw OR 'hay fever':ti,ab,kw OR allergy*:ti,ab,kw OR 'drug hypersensitivity':ti,ab,kw OR 'food allergy*':ti,ab,kw OR hypersensitivity*:ti,ab,kw) AND ('meta analysis'/exp OR 'meta analysis':ti,ab,kw OR 'meta analyses':ti,ab,kw OR 'systematic review'/exp OR 'systematic review*':ti,ab,kw)
Web of Science
TS=(Vitamin D OR 25-hydroxyvitamin D OR 25(OH)D OR VD) AND TS=(allergic disease* OR anaphylactic disease* OR hypersensitivity disease* OR atopic disease* OR asthma OR wheeze OR eczema OR urticaria OR atopic dermatitis OR allergic rhinitis* OR allergic rhinoconjunctivitis OR allergic conjunctivitis OR allergic purpura OR hay fever OR allergy* OR food allergy* OR drug hypersensitivity OR hypersensitivity) AND TS=(systematic review* OR meta analysis OR meta analyses)
Cochrane Database of Systematic Reviews
(Vitamin D OR VD) AND TS=(allergic disease* OR anaphylactic disease* OR hypersensitivity disease* OR atopic disease* OR asthma OR wheeze OR eczema OR urticaria OR atopic dermatitis OR allergic rhinitis* OR allergic rhinoconjunctivitis OR allergic conjunctivitis OR allergic purpura OR hay fever OR allergy* OR food allergy* OR drug hypersensitivity OR hypersensitivity)

The list of excluded articles by full text screening with exclusion reason

No.	Author, year	Reasons for exclusion
1	Autier 2017	It did not study the association between vitamin D (levels and supplementation) and allergic diseases.
2	Jolliffe 2021	It did not study the association between vitamin D (levels and supplementation) and allergic diseases.
3	Roth 2018	It did not study the association between vitamin D (levels and supplementation) and allergic diseases.
4	Vassilopoulou 2024	It did not study the association between vitamin D (levels and supplementation) and allergic diseases.
5	Psaroulaki 2023	It did not study the association between vitamin D (levels and supplementation) and allergic diseases.
6	Feng 2017	It did not study the association between vitamin D (levels and supplementation) and allergic diseases.
7	Yepes-Nuñez 2018	It did not provide sufficient data for re-analysis.
8	Venter 2020	It did not provide sufficient data for re-analysis.
9	Harvey 2014	It did not provide sufficient data for re-analysis.
10	Curtis 2018	It did not provide sufficient data for re-analysis.
11	Robison 2010	It did not provide sufficient data for re-analysis.
12	Mustapa 2020	It did not provide sufficient data for re-analysis.
13	Cornillier 2018	It did not provide sufficient data for re-analysis.
14	Fried 2016	It did not provide sufficient data for re-analysis.
15	Huang 2018	It did not provide sufficient data for re-analysis.
16	Tuchinda 2018	It did not provide sufficient data for re-analysis.

1. Autier P, Mullie P, Macacu A, et al. Effect of vitamin D supplementation on non-skeletal disorders: a systematic review of meta-analyses and randomised trials. Lancet Diabetes Endocrinol. 2017;5(12):986-1004. doi:10.1016/S2213-8587(17)30357-1
2. Jolliffe DA, Camargo CA Jr, Sluyter JD, et al. Vitamin D supplementation to prevent acute respiratory infections: a systematic review and meta-analysis of aggregate data from randomised controlled trials. Lancet Diabetes Endocrinol. 2021;9(5):276-292. doi:10.1016/S2213-8587(21)00051-6
3. Roth DE, Leung M, Mesfin E, Qamar H, Watterworth J, Papp E. Vitamin D supplementation during pregnancy: state of the evidence from a systematic review of randomised trials. BMJ. 2017;359:j5237. Published 2017 Nov 29. doi:10.1136/bmj.j5237
4. Vassilopoulou E, Comotti A, Douladiris N, et al. A systematic review and meta-analysis of nutritional and dietary interventions in randomized controlled trials for skin symptoms in children with atopic dermatitis and without food allergy: An EAACI task force report. Allergy. 2024;79(7):1708-1724. doi:10.1111/all.16160
5. Psaroulaki E, Katsaras GN, Samartzi P, et al. Association of food allergy in children with vitamin D insufficiency: a systematic review and meta-analysis. Eur J Pediatr. 2023;182(4):1533-1554. doi:10.1007/s00431-023-04843-2
6. Feng H, Xun P, Pike K, et al. In utero exposure to 25-hydroxyvitamin D and risk of childhood asthma, wheeze, and respiratory tract infections: A meta-analysis of birth cohort studies. J Allergy Clin Immunol. 2017;139(5):1508-1517. doi:10.1016/j.jaci.2016.06.065
7. Yepes-Nuñez JJ, Brożek JL, Fiocchi A, et al. Vitamin D supplementation in primary allergy prevention: Systematic review of randomized and non-randomized studies. Allergy. 2018;73(1):37-49. doi:10.1111/all.13241
8. Venter C, Agostoni C, Arshad SH, et al. Dietary factors during pregnancy and atopic outcomes in childhood: A systematic review from the European Academy of Allergy and Clinical Immunology. Pediatr Allergy Immunol. 2020;31(8):889-912. doi:10.1111/pai.13303
9. Harvey NC, Holroyd C, Ntani G, et al. Vitamin D supplementation in pregnancy: a systematic review. Health Technol Assess. 2014;18(45):1-190. doi:10.3310/hta18450
10. Curtis EM, Moon RJ, Harvey NC, Cooper C. Maternal vitamin D supplementation during pregnancy. British medical bulletin. 2018 Jun 1;126(1):57-77.
11. Robison R, Kumar R. The effect of prenatal and postnatal dietary exposures on childhood development of atopic disease. Curr Opin Allergy Clin Immunol. 2010;10(2):139-144. doi:10.1097/ACI.0b013e32833667a8
12. Mustapa Kamal Basha MA, Majid HA, Razali N, Yahya A. Risk of eczema, wheezing and respiratory tract infections in the first year of life: A systematic review of vitamin D concentrations during pregnancy and at birth. PLoS One. 2020;15(6):e0233890. Published 2020 Jun 15. doi:10.1371/journal.pone.0233890
13. Cornillier H, Giraudeau B, Munck S, et al. Chronic spontaneous urticaria in children - a systematic review on interventions and comorbidities. Pediatr Allergy Immunol. 2018;29(3):303-310. doi:10.1111/pai.12870
14. Fried DA, Rhyu J, Odato K, Blunt H, Karagas MR, Gilbert-Diamond D. Maternal and cord blood vitamin D status and childhood infection and allergic disease: a systematic review. Nutr Rev. 2016;74(6):387-410. doi:10.1093/nutrit/nuv108
15. Huang CM, Lara-Corrales I, Pope E. Effects of Vitamin D levels and supplementation on atopic dermatitis: A systematic review. Pediatr Dermatol. 2018;35(6):754-760. doi:10.1111/pde.13639
16. Tuchinda P, Kulthanan K, Chularojanamontri L, Arunkajohnsak S, Sriussadaporn S. Relationship between vitamin D and chronic spontaneous urticaria: a systematic review. Clin Transl Allergy. 2018;8:51. Published 2018 Dec 4. doi:10.1186/s13601-018-0234-7

The list of excluded overlapping and outdated articles

No.	Author, year	Reasons for exclusion
1	Kawada 2025	Another meta-analysis was selected as the eligible meta-analysis
2	Hidayati 2023	Another meta-analysis was selected as the eligible meta-analysis
3	Fu 2022	Another meta-analysis was selected as the eligible meta-analysis
4	Park 2023	Another meta-analysis was selected as the eligible meta-analysis
5	Hao 2022	Another meta-analysis was selected as the eligible meta-analysis
6	Wang 2022	Another meta-analysis was selected as the eligible meta-analysis
7	El Abd 2024	Another meta-analysis was selected as the eligible meta-analysis
8	Fedora 2024	Another meta-analysis was selected as the eligible meta-analysis
9	Sobczak 2023	Another meta-analysis was selected as the eligible meta-analysis
10	Pojsupap 2015	Another meta-analysis was selected as the eligible meta-analysis
11	Liu 2022	Another meta-analysis was selected as the eligible meta-analysis
12	Chen 2021	Another meta-analysis was selected as the eligible meta-analysis
13	El Abd 2024	Another meta-analysis was selected as the eligible meta-analysis
14	Shi 2021	Another meta-analysis was selected as the eligible meta-analysis
15	Li 2025	Another meta-analysis was selected as the eligible meta-analysis
16	Shen 2025	Another meta-analysis was selected as the eligible meta-analysis
17	Li 2022	Another meta-analysis was selected as the eligible meta-analysis
18	Wei 2016	This article is outdated.
19	Beckhaus 2015	This article is outdated.
20	Kim 2016	This article is outdated.
21	Aryan 2017	This article is outdated.
22	Kim 2016	This article is outdated.
23	Hattangdi-Haridas 2019	This article is outdated.
24	Kim 2016	This article is outdated.
25	Zhu 2019	This article is outdated.
26	Jolliffe 2017	This article is outdated.
27	Riverin 2015	This article is outdated.
28	Wolsk 2017	This article is outdated.
29	Luo 2015	This article is outdated.
30	Vahdaninia 2017	This article is outdated.
31	Li 2019	This article is outdated.
32	Christensen 2017	This article is outdated.
33	Jat 2017	This article is outdated.
34	Wang 2019	This article is outdated.
35	Zhang 2014	This article is outdated.
36	Fares 2015	This article is outdated.
37	Song 2017	This article is outdated.
38	Shen 2018	This article is outdated.
39	Wang 2018	This article is outdated.

1. Kawada K, Sato C, Ishida T, et al. Vitamin D Supplementation and Allergic Rhinitis: A Systematic Review and Meta-Analysis. Medicina (Kaunas). 2025;61(2):355. Published 2025 Feb 18. doi:10.3390/medicina61020355
2. Hidayati AN, Sawitri S, Sari DW, et al. Efficacy of vitamin D supplementation on the severity of atopic dermatitis in children: A systematic review and meta-analysis. F1000Res. 2023;11:274. Published 2023 Sep 25. doi:10.12688/f1000research.106957.2
3. Fu H, Li Y, Huang H, Wang D. Serum Vitamin D Level and Efficacy of Vitamin D Supplementation in Children with Atopic Dermatitis: A Systematic Review and Meta-analysis. Comput Math Methods Med. 2022;2022:9407888. Published 2022 Jul 20. doi:10.1155/2022/9407888
4. Park JS, Kim M, Sol IS, et al. Effect of Vitamin D on the Treatment of Atopic Dermatitis With Consideration of Heterogeneities: Meta-Analysis of Randomized Controlled Trials. Allergy Asthma Immunol Res. 2023;15(2):262-270. doi:10.4168/aair.2023.15.2.262
5. Hao M, Xu R, Luo N, Liu M, Xie J, Zhang W. The Effect of Vitamin D Supplementation in Children With Asthma: A Meta-Analysis. Front Pediatr. 2022;10:840617. Published 2022 Jun 29. doi:10.3389/fped.2022.840617
6. Wang Y, Wang J, Chen L, et al. Efficacy of vitamin D supplementation on COPD and asthma control: A systematic review and meta-analysis. J Glob Health. 2022;12:04100. Published 2022 Dec 16. doi:10.7189/jogh.12.04100
7. El Abd A, Dasari H, Dodin P, Trottier H, Ducharme FM. The effects of vitamin D supplementation on inflammatory biomarkers in patients with asthma: a systematic review and meta-analysis of randomized controlled trials. Front Immunol. 2024;15:1335968. Published 2024 Mar 13. doi:10.3389/fimmu.2024.1335968
8. Fedora K, Setyoningrum RA, Aina Q, Rosyidah LN, Ni'mah NL, Titiharja FF. Vitamin D supplementation decrease asthma exacerbations in children: a

systematic review and meta-analysis of randomized controlled trials. Ann Med. 2024;56(1):2400313. doi:10.1080/07853890.2024.2400313
9. Sobczak M, Pawliczak R. Relationship between vitamin D and asthma from gestational to adulthood period: a meta-analysis of randomized clinical trials. BMC Pulm Med. 2023;23(1):212. Published 2023 Jun 17. doi:10.1186/s12890-023-02514-4
10. Pojsupap S, Iliriani K, Sampaio TZ, et al. Efficacy of high-dose vitamin D in pediatric asthma: a systematic review and meta-analysis. J Asthma. 2015;52(4):382-390. doi:10.3109/02770903.2014.980509
11. Liu M, Wang J, Sun X. A Meta-Analysis on Vitamin D Supplementation and Asthma Treatment. Front Nutr. 2022;9:860628. Published 2022 Jul 6. doi:10.3389/fnut.2022.860628
12. Chen Z, Peng C, Mei J, Zhu L, Kong H. Vitamin D can safely reduce asthma exacerbations among corticosteroid-using children and adults with asthma: a systematic review and meta-analysis of randomized controlled trials. Nutr Res. 2021;92:49-61. doi:10.1016/j.nutres.2021.05.010
13. El Abd A, Dasari H, Dodin P, Trottier H, Ducharme FM. Associations between vitamin D status and biomarkers linked with inflammation in patients with asthma: a systematic review and meta-analysis of interventional and observational studies. Respir Res. 2024;25(1):344. Published 2024 Sep 19. doi:10.1186/s12931-024-02967-z
14. Shi D, Wang D, Meng Y, Chen J, Mu G, Chen W. Maternal vitamin D intake during pregnancy and risk of asthma and wheeze in children: a systematic review and meta-analysis of observational studies. J Matern Fetal Neonatal Med. 2021;34(4):653-659. doi:10.1080/14767058.2019.1611771
15. Li Q, Xu X, Liu Y, et al. The effects of prenatal vitamin D supplementation on respiratory and allergy-related outcomes in children: A systematic review and meta-analysis of randomized controlled trials. World Allergy Organ J. 2025;18(7):101075. Published 2025 Jun 7. doi:10.1016/j.waojou.2025.101075
16. Shen J, Zhou Z, Feng F, Zhang Y, Bao S, Feng Y. Association between prenatal vitamin D supplementation and respiratory diseases in children: a systematic review and meta-analysis. BMC Pediatr. 2025;25(1):915. Published 2025 Nov 7. doi:10.1186/s12887-025-06268-2
17. Li Q, Zhou Q, Zhang G, et al. Vitamin D Supplementation and Allergic Diseases during Childhood: A Systematic Review and Meta-Analysis. Nutrients. 2022;14(19):3947. Published 2022 Sep 23. doi:10.3390/nu14193947
18. Wei Z, Zhang J, Yu X. Maternal vitamin D status and childhood asthma, wheeze, and eczema: A systematic review and meta-analysis. Pediatr Allergy Immunol. 2016;27(6):612-619. doi:10.1111/pai.12593
19. Beckhaus AA, Garcia-Marcos L, Forno E, Pacheco-Gonzalez RM, Celedón JC, Castro-Rodriguez JA. Maternal nutrition during pregnancy and risk of asthma, wheeze, and atopic diseases during childhood: a systematic review and meta-analysis. Allergy. 2015;70(12):1588-1604. doi:10.1111/all.12729
20. Kim YH, Kim KW, Kim MJ, et al. Vitamin D levels in allergic rhinitis: a systematic review and meta-analysis. Pediatr Allergy Immunol. 2016;27(6):580-590. doi:10.1111/pai.12599
21. Aryan Z, Rezaei N, Camargo CA Jr. Vitamin D status, aeroallergen sensitization, and allergic rhinitis: A systematic review and meta-analysis. Int Rev Immunol. 2017;36(1):41-53. doi:10.1080/08830185.2016.1272600
22. Kim MJ, Kim SN, Lee YW, Choe YB, Ahn KJ. Vitamin D Status and Efficacy of Vitamin D Supplementation in Atopic Dermatitis: A Systematic Review and Meta-Analysis. Nutrients. 2016;8(12):789. Published 2016 Dec 3. doi:10.3390/nu8120789
23. Hattangdi-Haridas SR, Lanham-New SA, Wong WHS, Ho MHK, Darling AL. Vitamin D Deficiency and Effects of Vitamin D Supplementation on Disease Severity in Patients with Atopic Dermatitis: A Systematic Review and Meta-Analysis in Adults and Children. Nutrients. 2019;11(8):1854. Published 2019 Aug 9. doi:10.3390/nu11081854
24. Kim G, Bae JH. Vitamin D and atopic dermatitis: A systematic review and meta-analysis. Nutrition. 2016;32(9):913-920. doi:10.1016/j.nut.2016.01.023
25. Zhu Z, Yang Z, Wang C, Liu H. Assessment of the Effectiveness of Vitamin Supplement in Treating Eczema: A Systematic Review and Meta-Analysis. Evid Based Complement Alternat Med. 2019;2019:6956034. Published 2019 Oct 31. doi:10.1155/2019/6956034
26. Jolliffe DA, Greenberg L, Hooper RL, et al. Vitamin D supplementation to prevent asthma exacerbations: a systematic review and meta-analysis of individual participant data. Lancet Respir Med. 2017;5(11):881-890. doi:10.1016/S2213-2600(17)30306-5
27. Riverin BD, Maguire JL, Li P. Vitamin D Supplementation for Childhood Asthma: A Systematic Review and Meta-Analysis. PLoS One. 2015;10(8):e0136841. Published 2015 Aug 31. doi:10.1371/journal.pone.0136841
28. Wolsk HM, Chawes BL, Litonjua AA, et al. Prenatal vitamin D supplementation reduces risk of asthma/recurrent wheeze in early childhood: A combined analysis of two randomized controlled trials. PLoS One. 2017;12(10):e0186657. Published 2017 Oct 27. doi:10.1371/journal.pone.0186657
29. Luo J, Liu D, Liu CT. Can Vitamin D Supplementation in Addition to Asthma Controllers Improve Clinical Outcomes in Patients With Asthma?: A Meta-Analysis. Medicine (Baltimore). 2015;94(50):e2185. doi:10.1097/MD.0000000000002185
30. Vahdaninia M, Mackenzie H, Helps S, Dean T. Prenatal Intake of Vitamins and Allergic Outcomes in the Offspring: A Systematic Review and Meta-Analysis. J Allergy Clin Immunol Pract. 2017;5(3):771-778.e5. doi:10.1016/j.jaip.2016.09.024
31. Li W, Qin Z, Gao J, et al. Vitamin D supplementation during pregnancy and the risk of wheezing in offspring: a systematic review and dose-response meta-analysis. J Asthma. 2019;56(12):1266-1273. doi:10.1080/02770903.2018.1536142
32. Christensen N, Søndergaard J, Fisker N, Christesen HT. Infant Respiratory Tract Infections or Wheeze and Maternal Vitamin D in Pregnancy: A Systematic Review. Pediatr Infect Dis J. 2017;36(4):384-391. doi:10.1097/INF.0000000000001452
33. Jat KR, Khairwa A. Vitamin D and asthma in children: A systematic review and meta-analysis of observational studies. Lung India. 2017;34(4):355-363. doi:10.4103/0970-2113.209227
34. Wang M, Liu M, Wang C, et al. Association between vitamin D status and asthma control: A meta-analysis of randomized trials. Respir Med. 2019;150:85-94. doi:10.1016/j.rmed.2019.02.016
35. Zhang LL, Gong J, Liu CT. Vitamin D with asthma and COPD: not a false hope? A systematic review and meta-analysis. Genet Mol Res. 2014;13(3):7607-7616. Published 2014 Feb 13. doi:10.4238/2014.February.13.10
36. Fares MM, Alkhaled LH, Mroueh SM, Akl EA. Vitamin D supplementation in children with asthma: a systematic review and meta-analysis. BMC Res Notes. 2015;8:23. Published 2015 Feb 3. doi:10.1186/s13104-014-0961-3
37. Song H, Yang L, Jia C. Maternal vitamin D status during pregnancy and risk of childhood asthma: A meta-analysis of prospective studies. Mol Nutr Food Res. 2017;61(5):10.1002/mnfr.201600657. doi:10.1002/mnfr.201600657
38. Shen SY, Xiao WQ, Lu JH, et al. Early life vitamin D status and asthma and wheeze: a systematic review and meta-analysis. BMC Pulm Med. 2018;18(1):120. Published 2018 Jul 20. doi:10.1186/s12890-018-0679-4
39. Wang X, Li X, Shen Y, Wang X. The association between serum vitamin D levels and urticaria: a meta-analysis of observational studies. G Ital Dermatol Venereol. 2018;153(3):389-395. doi:10.23736/S0392-0488.17.05774-1

Reviews with overlapping primary studies

Mark with a "1" each intersectionrepresenting a primary study includedin a systematic review.

Table S1: Vitamin D supplementation in allergic rhinitis

Primary Studies	Systematic Reviews	
Study ID	Surayya 2025	Kawada 2025
Columbo 2014	1	
Modh 2014	1	
jerzynska 2016	1	1
Menon 2016	1	
Gupta 2017	1	
Handoko 2017	1	
Reddy 2018	1	
Agarwal 2019	1	
Bakhshaee 2019	1	1
EI Maghraby 2019	1	
Velankar 2019	1	
Bhardwaj 2020	1	
Liu 2020	1	
Kalsotra 2022	1	
Guo 2023		1
Bhardwaj 2021		1
Chiewchalermsri 2024		1
**Number of columns (number of reviews)**	**c**	**2**
**Number of rows (number of index publications)**	**r**	**17**
**Number of included primary studies (including double counting)**	**N**	**19**
**Covered area**	**N/(rc)**	**55.88%**
**Corrected covered area**	**(N-r)/(rc-r)**	**11.76%**
**Interpretation of overlap**	**Very High overlap**	

Table S2: Prenatal vitamin D supplementation and offspring allergic rhinitis

Primary Studies	Systematic Reviews	
Study ID	Luo 2022	Tareke 2020
Brustad 2019	1	1
Goldring 2013	1	1
Litonjua 2020	1	1
**Number of columns (number of reviews)**	**c**	**2**
**Number of rows (number of index publications)**	**r**	**3**
**Number of included primary studies (including double counting)**	**N**	**6**
**Covered area**	**N/(rc)**	**100.00%**
**Corrected covered area**	**(N-r)/(rc-r)**	**100.00%**
**Interpretation of overlap**	**Very High overlap**	

Table S3: Vitamin D supplementation in atopic dermatitis

**Primary Studies**	**Systematic Reviews**						
**Study ID**	**Hidayati 2023**	**Fu 2022**	**Chun Ng 2022**	**Park 2023**	**Nielsen 2024**	**Li 2022**	**Luo 2022**
Camargo 2014	1	1			1	1	
Armendariz 2018	1			1	1		
Corrales 2018	1		1	1	1	1	
Earlia 2020	1					1	
Di Fillippo 2015		1	1				
Raj 2020		1					
Amestejani 2012		1			1		
Javanbakht 2011		1	1	1	1		
Lara 2019		1					
Mansour 2020		1			1	1	
Sidbury 2008		1			1	1	
Samochocki 2013						1	
Udompataikul 2015			1	1	1	1	
Galli 2015					1	1	
Noha 2020					1		
Modi 2021					1		
Borzutzky 2024					1		
Rosendahi 2019							1
Rueter 2018							1
Bäck 2009							1
**Number of columns (number of reviews)**			**c**		**7**		
**Number of rows (number of index publications)**			**r**		**20**		
**Number of included primary studies (including double counting)**			**N**		**43**		
**Covered area**			**N/(rc)**		**30.71%**		
**Corrected covered area**			**(N-r)/(rc-r)**		**19.17%**		
**Interpretation of overlap**			**Very High overlap**				

Table S4: Serum 25 (OH) D levels and atopic dermatitis

**Primary Studies**	**Systematic Reviews**	
Study ID	**Fu 2022**	**Chun Ng 2022**
Ahmed 2021	1	
Cheon 2015	1	1
DAuria 2017	1	1
Daniluk 2019	1	
Dogru 2018	1	
El Taieb 2013	1	1
Lee 2019	1	
Lipinska 2021	1	
Machura 2018	1	
Sanmartin 2020	1	1
Sharma 2017	1	1
Su 2017	1	1
Wang 2014	1	1
Xiang 2019	1	1
Di Filippo 2015		1
Hata 2014		1
Noh 2014		1
Amon 2018		1
Farajzadeh 2015		1
Mohamed 2019		1
**Number of columns (number of reviews)**	**c**	**2**
**Number of rows (number of index publications)**	**r**	**20**
**Number of included primary studies (including double counting)**	**N**	**28**
**Covered area**	**N/(rc)**	**70.00%**
**Corrected covered area**	**(N-r)/(rc-r)**	**40.00%**
**Interpretation of overlap**	**Very High overlap**	

Table S5: Prenatal vitamin D supplementation and offspring atopic dermatitis

Primary Studies	Systematic Reviews			
Study ID	Venter 2020	Luo 2022	Li 2025	Shen 2025
Goldring 2013	1	1	1	1
Litonjua 2016	1	1	1	1
Chawes 2016	1	1	1	1
**Number of columns (number of reviews)**		**c**	**4**	
**Number of rows (number of index publications)**		**r**	**3**	
**Number of included primary studies (including double counting)**		**N**	**12**	
**Covered area**		**N/(rc)**	**100.00%**	
**Corrected covered area**		**(N-r)/(rc-r)**	**100.00%**	
**Interpretation of overlap**		**Very High overlap**		

Table S6: Vitamin D supplementation and asthma

Primary Studies	Systematic Reviews										
**Study ID**	**Hao 2022**	**Wang 2022**	**Wang 2021**	**Liu 2022**	**Chen 2021**	**Williamson 2023**	**Li 2022**	**Sobczak 2023**	**Asmae El Abd 2024**	**Fedora 2024**	**Wang 2023**
Bar 2015	1								1		
Duchharme 2019	1				1	1	1				
Forno 2020	1	1			1	1				1	
Jat 2021	1	1				1				1	
Kerley 2016	1		1			1	1				
Thakur 2021	1			1		1				1	
Anduiar-Espinosa 2020		1			1				1		
Arshi 2014		1									
Castro 2014		1		1	1	1					
Dodamani 2019		1		1	1						
Jensen 2016		1				1					
Majak 2011		1	1	1	1	1	1			1	
Martineau 2015		1		1	1	1					
Tachimoto 2016		1	1			1	1			1	
Hakur 2021		1									
Kan 2018			1							1	
Yadav 2014			1	1	1		1			1	
Musharraf 2017				1							
Camargo 2021						1					
Jerzynska 2016						1					

Majak 2009						1					
Urashima 2010			1			1	1			1	
Alansari 2017							1				
DIVA 2016							1				
ESDAC 2021							1				
DKA 2020							1				
ViDASTA 2021							1				
Rosendahl 2019								1			
Hibbs 2018								1			
Rueter 2020								1			
Litonjua 2020			1								
Romas 2018									1		
Rosser 2022									1		
De Groot 2015									1		
Back 2009											1
Hypponen 2004											1
Nwaru 2017								1			1
**Number of columns (number of reviews)**						**c**			**11**		
**Number of rows (number of index publications)**						**r**			**37**		
**Number of included primary studies (including double counting)**						**N**			**84**		
**Covered area**						**N/(rc)**			**20.64%**		
**Corrected covered area**						**(N-r)/(rc-r)**			**12.70%**		
**Interpretation of overlap**						**High overlap**					

Table S7: Prenatal vitamin D supplementation and offspring asthma

Primary Studies	Systematic Reviews						
Study ID	Tareke 2020	Sobczak 2023	Shi 2021	Venter 2020	Luo 2022	Li 2025	Shen 2025
Goldring 2013	1			1	1	1	1
Grant 2016	1						
Brustad 2019	1	1			1		
Litonjua 2016	1			1		1	1
Chaws 2016	1	1		1	1	1	1
Litonjua 2020	1	1			1		
Magnus 2013			1				
Tolppanen 2013			1				
Allan 2015			1				
Pike 2012			1				
Maslova 2013			1				
Erkkola 2009			1				
Anderson 2015			1				
Gale 2008				1			
**Number of columns (number of reviews)**			**c**		**7**		
**Number of rows (number of index publications)**			**r**		**14**		
**Number of included primary studies (including double counting)**			**N**		**30**		
**Covered area**			**N/(rc)**		**30.61%**		
**Corrected covered area**			**(N-r)/(rc-r)**		**19.05%**		
**Interpretation of overlap**			**Very High overlap**				

Table S8: The results of the AMSTAR-2 assessment

Included studies	AMSTAR-2																Overall quality
	Q1	Q2	Q3	Q4	Q5	Q6	Q7	Q8	Q9	Q10	Q11	Q12	Q13	Q14	Q15	Q16	
Ju 2023[30]	Y	N	Y	PY	Y	Y	PY	Y	Y	Y	Y	Y	Y	Y	Y	Y	Low
Pacheco 2018[31]	Y	PY	Y	PY	Y	Y	PY	Y	Y	Y	Y	N	N	N	Y	Y	Low
Ng 2022[32]	Y	PY	Y	PY	Y	Y	Y	Y	Y	Y	Y	Y	Y	Y	Y	Y	High
Hidajat 2024[33]	Y	Y	Y	PY	Y	Y	PY	Y	Y	Y	Y	Y	N	N	N	Y	Critically low
Wang 2021[34]	Y	PY	Y	PY	Y	Y	PY	Y	Y	Y	Y	Y	Y	N	N	Y	Low
Li 2021[35]	Y	Y	Y	PY	Y	Y	PY	Y	Y	Y	Y	Y	Y	Y	Y	Y	High
Daneshvar2024[36]	Y	PY	Y	PY	Y	Y	PY	Y	Y	Y	Y	Y	Y	Y	Y	Y	High
Zheng 2025[37]	Y	PY	Y	PY	Y	Y	PY	Y	Y	Y	Y	N	Y	Y	Y	Y	Moderate
Luo 2022[38]	Y	PY	Y	PY	Y	Y	PY	Y	Y	Y	Y	N	Y	Y	Y	Y	Critically low
Surayya 2025[39]	Y	Y	Y	PY	Y	Y	PY	Y	Y	Y	Y	Y	Y	N	Y	Y	Moderate
Nielsen 2024[40]	Y	Y	Y	PY	Y	Y	PY	Y	Y	Y	Y	Y	Y	Y	Y	Y	High
Tareke 2020[41]	Y	PY	Y	PY	Y	Y	PY	Y	Y	Y	Y	Y	N	N	Y	Y	Low
Wang 2023[42]	Y	Y	Y	PY	Y	Y	PY	Y	Y	Y	Y	Y	Y	N	Y	Y	Moderate
Williamson 2023[43]	Y	Y	Y	PY	Y	Y	PY	Y	Y	Y	Y	Y	Y	Y	Y	Y	High

Y, yes; PY, partial yes; N, no.

Q1: Do the research issue and inclusion standard reviewed include component parts of PICO?

Q2: Do the authors report includes a clear statement of the methodology was established before review, and whether it showed any major deviation from the
agreement?

Q3: Do the authors describe the selection of inclusion research design?

Q4: Do the authors adopt synthetical document retrieval strategy?

Q5: Whether two authors independently screen articles?

Q6: Do two authors extract data independently?

Q7: Do the authors provide a list and reasons for excluding studies?

Q8: Do the authors detailly describe the brought into studies?

Q9: Do the authors use appropriate techniques to evaluate the risk of bias for separate studies?

Q10: Have the authors reported the financial sources for the research listed?

Q11: If conducted meta-analysis, does the author combined the results in an appropriate way?

Q12: If conducted meta-analysis, does the author evaluate the potent influence of study bias on meta-analysis results or other comprehensive evidence?

Q13: When in the discussion of results, did the authors explain the role of bias in the individual study?

Q14: Does the author offer a reasonable interpretation and discussion for the heterogenicity observed in the outcome?

Q15: If quantity summary was made, whether the author examined publication bias sufficiently and discussed its possible influence?

Q16: Has the author make known any possible conflicts of interest, any funds they get to conduct the study?

Figure S1. Random-effects meta-analysis of the association between serum 25 (OH) D level and allergic rhinitis (forest plot and funnel plot)

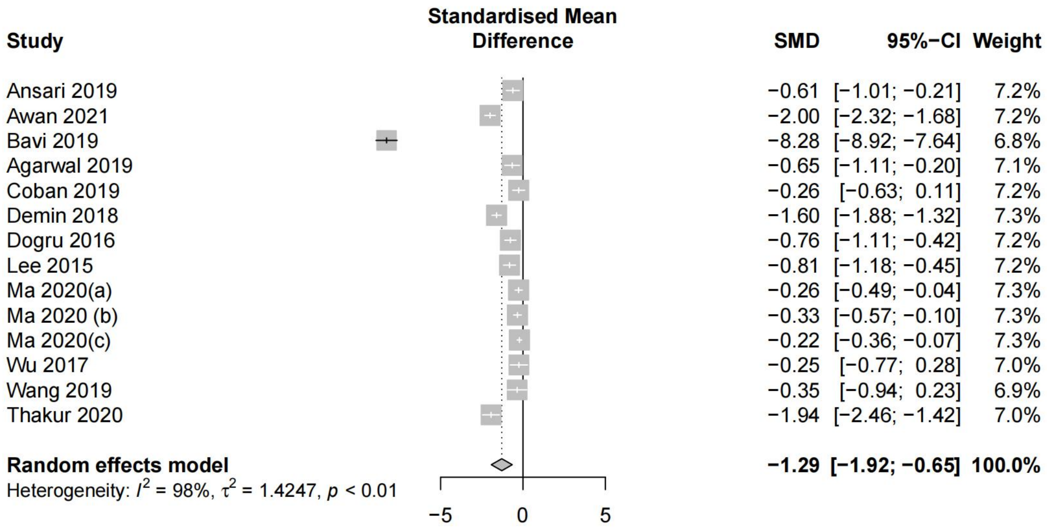

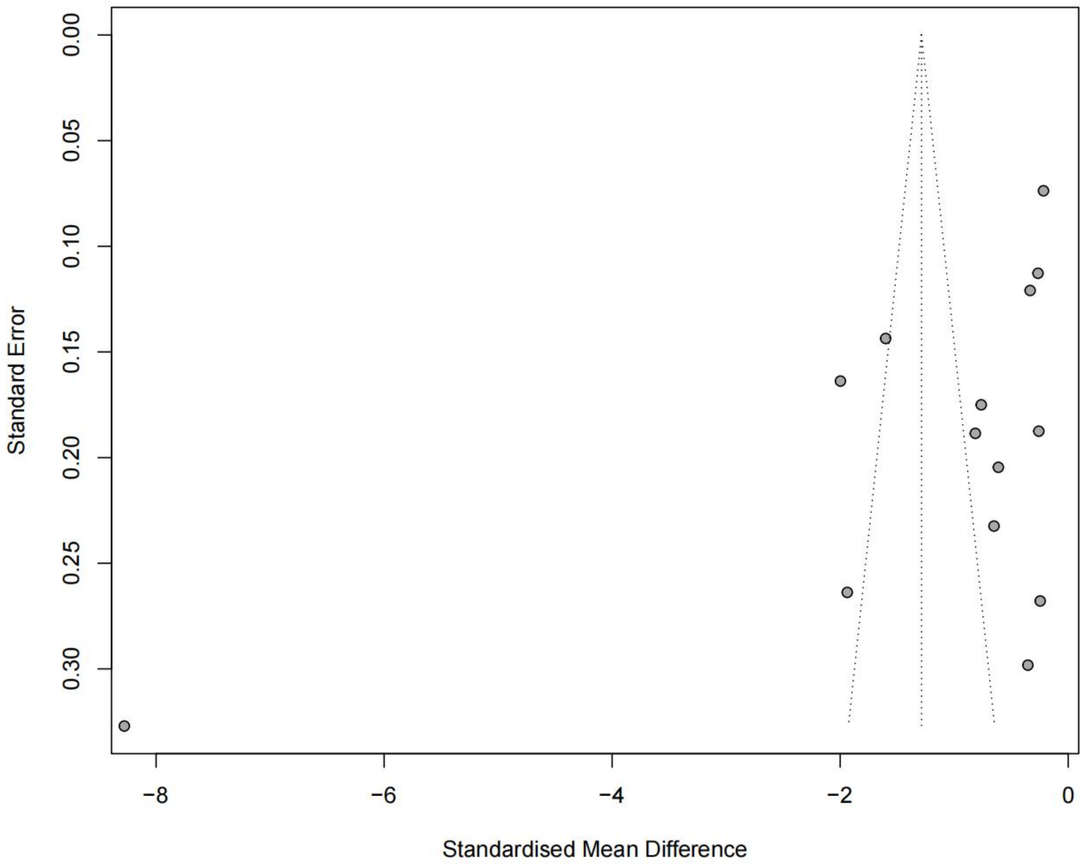

Figure S2. Random-effects meta-analysis of the association between serum 25 (OH) D level and atopic dermatitis (forest plot and funnel plot)

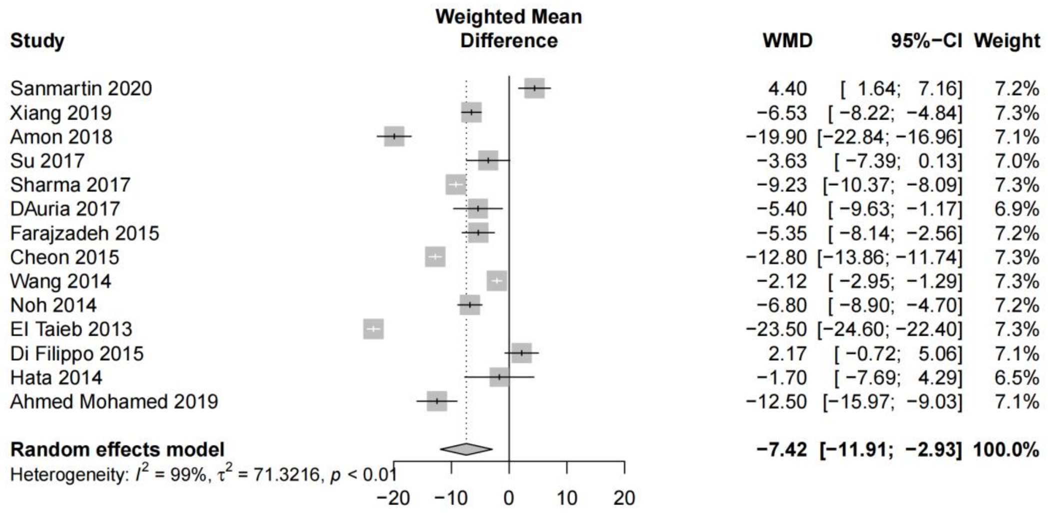

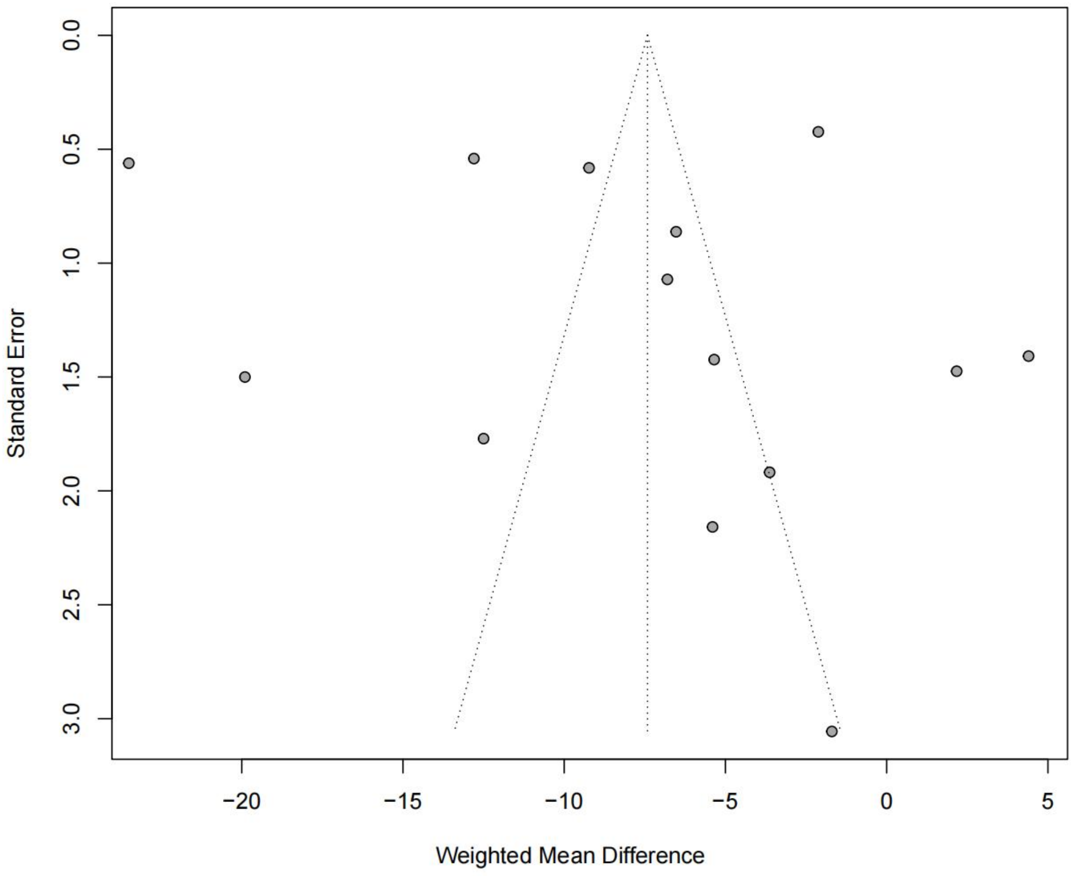

Figure S3. Random-effects meta-analysis of the association between serum 25 (OH) D level and asthma (forest plot and funnel plot)

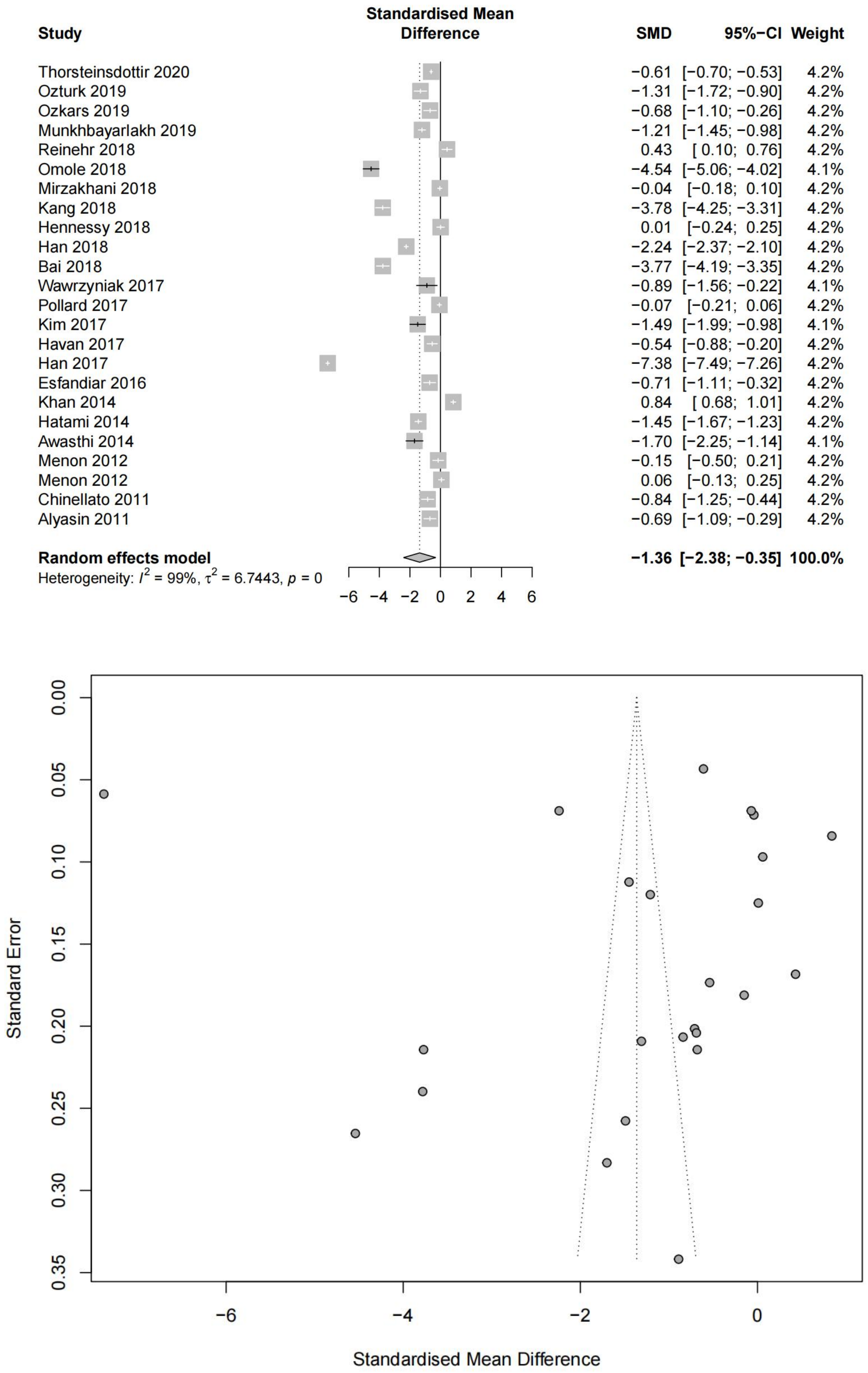

Figure S4. Random-effects meta-analysis of the association between serum 25 (OH) D level and urticaria (forest plot and funnel plot)

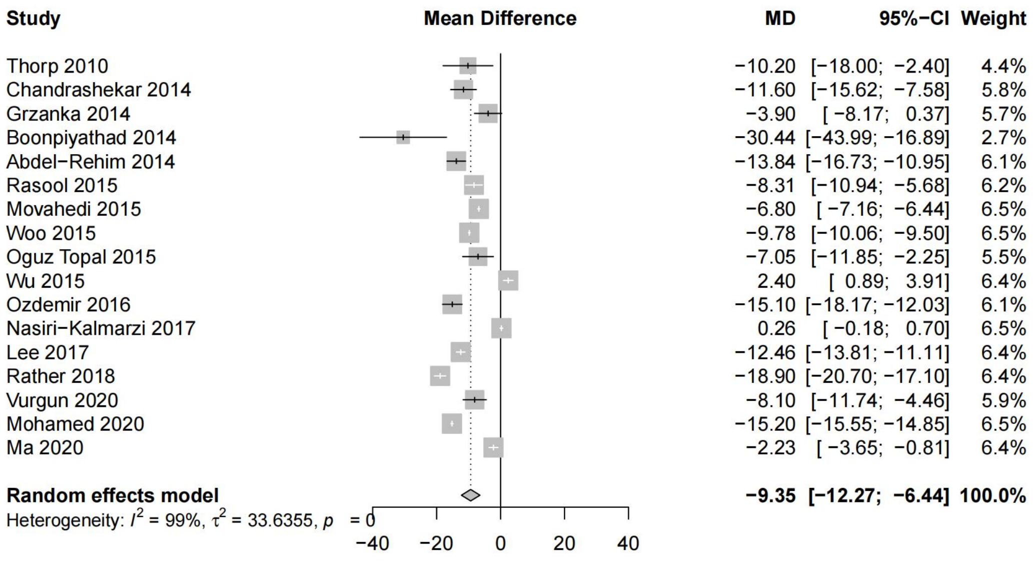

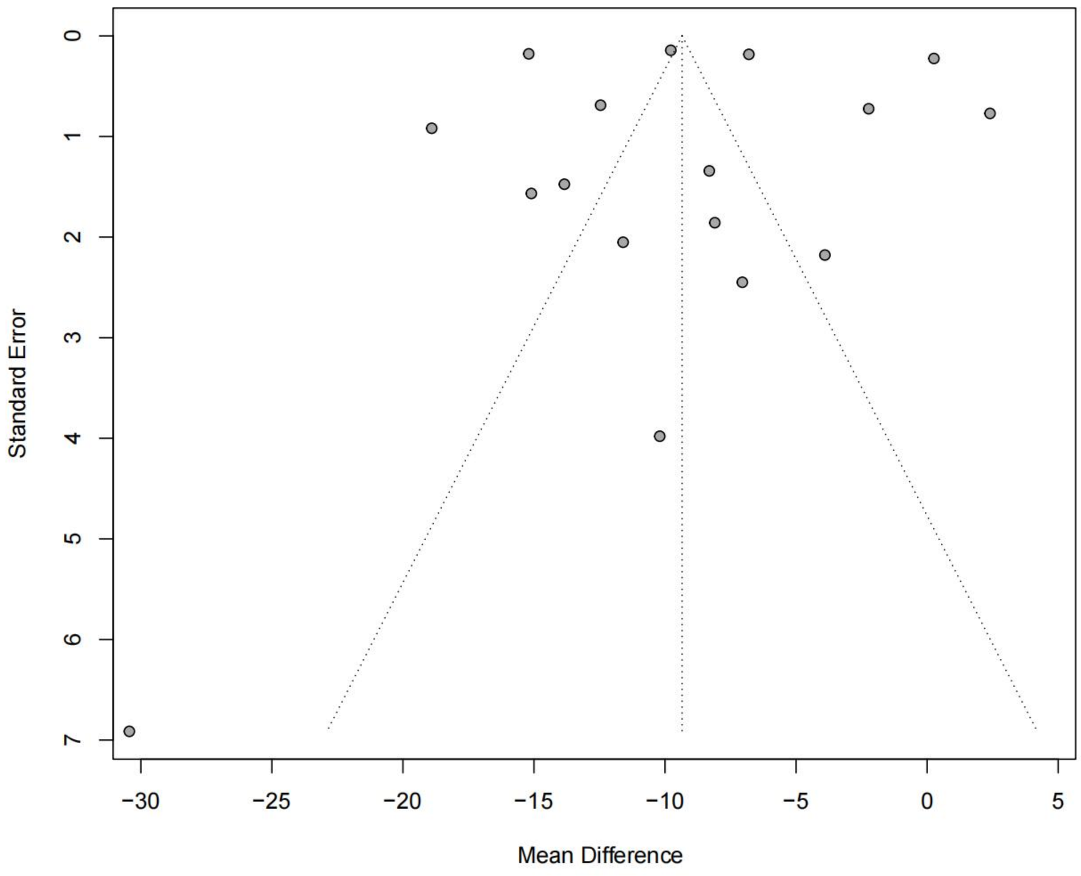

Figure S5. Random-effects meta-analysis of the association between serum 25 (OH) D level and vernal keratoconjunctivitis (forest plot and funnel plot)

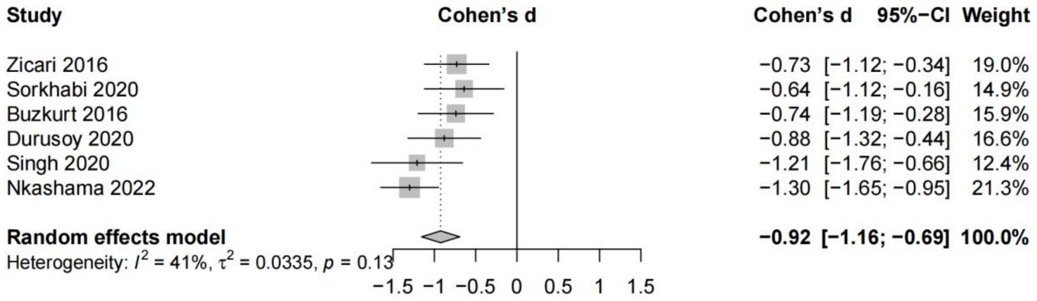

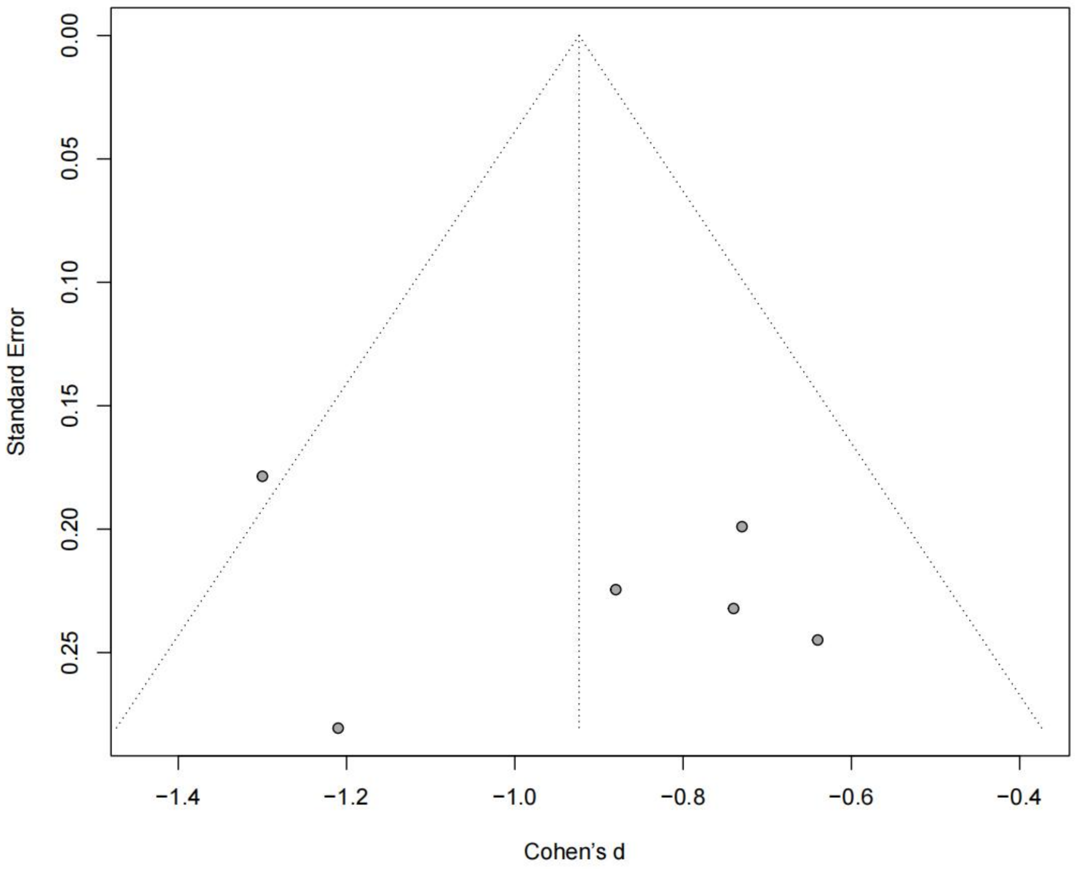

Figure S6. Random-effects meta-analysis of the association between serum 25 (OH) D level and cow's milk protein allergy (forest plot and funnel plot)

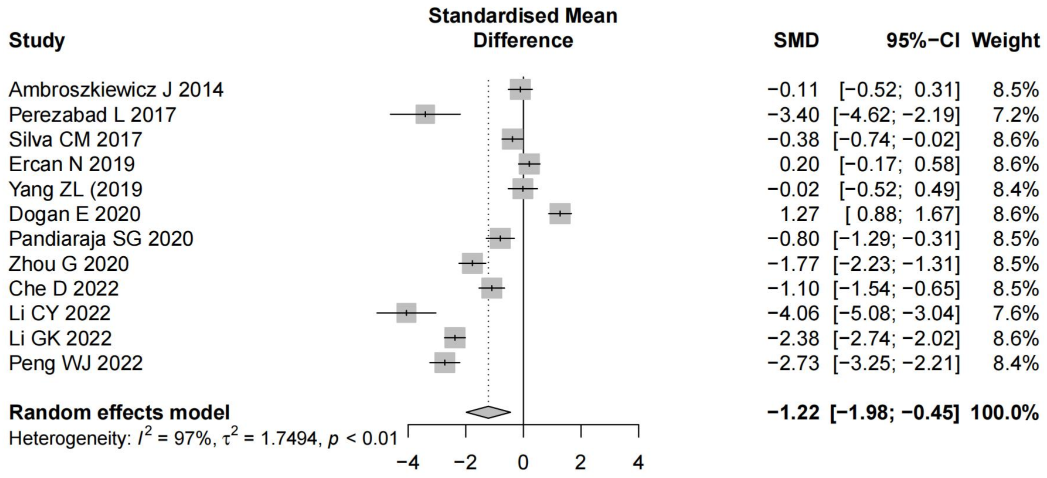

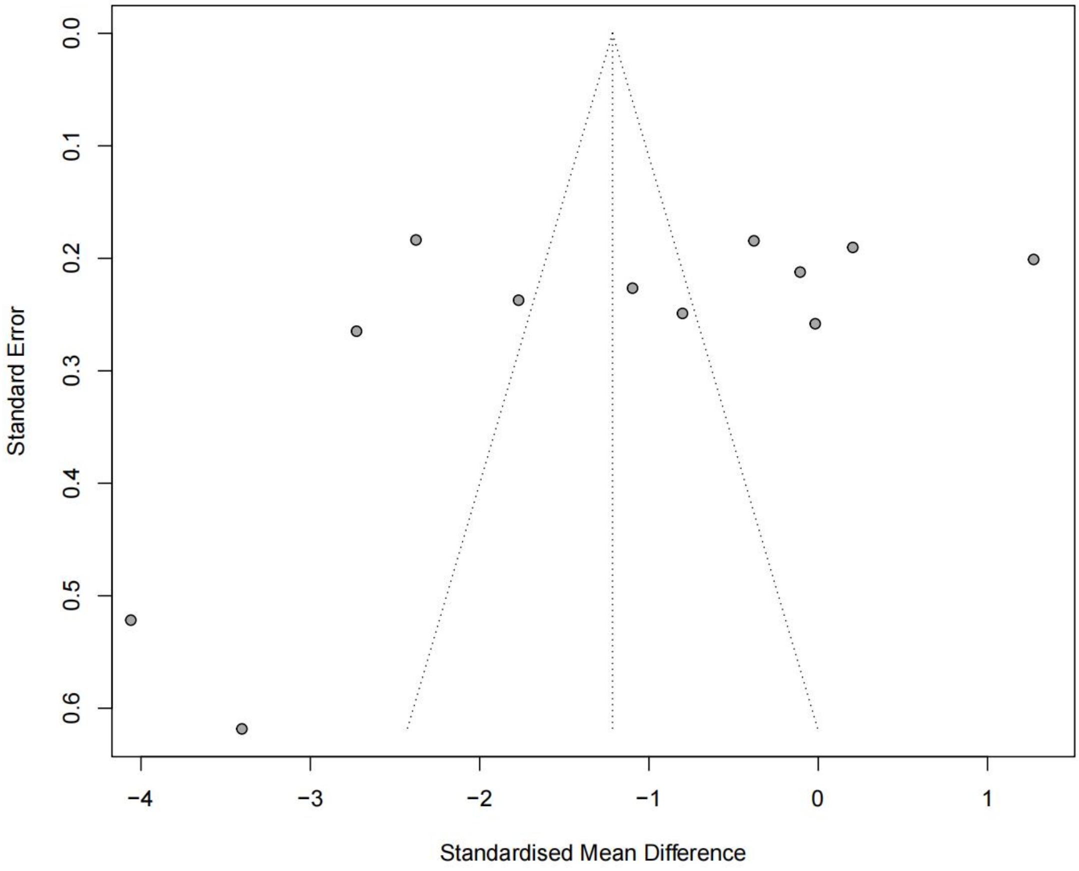

Figure S7. Random-effects meta-analysis of the association between prenatal vitamin D level and offspring allergic rhinitis (forest plot and funnel plot)

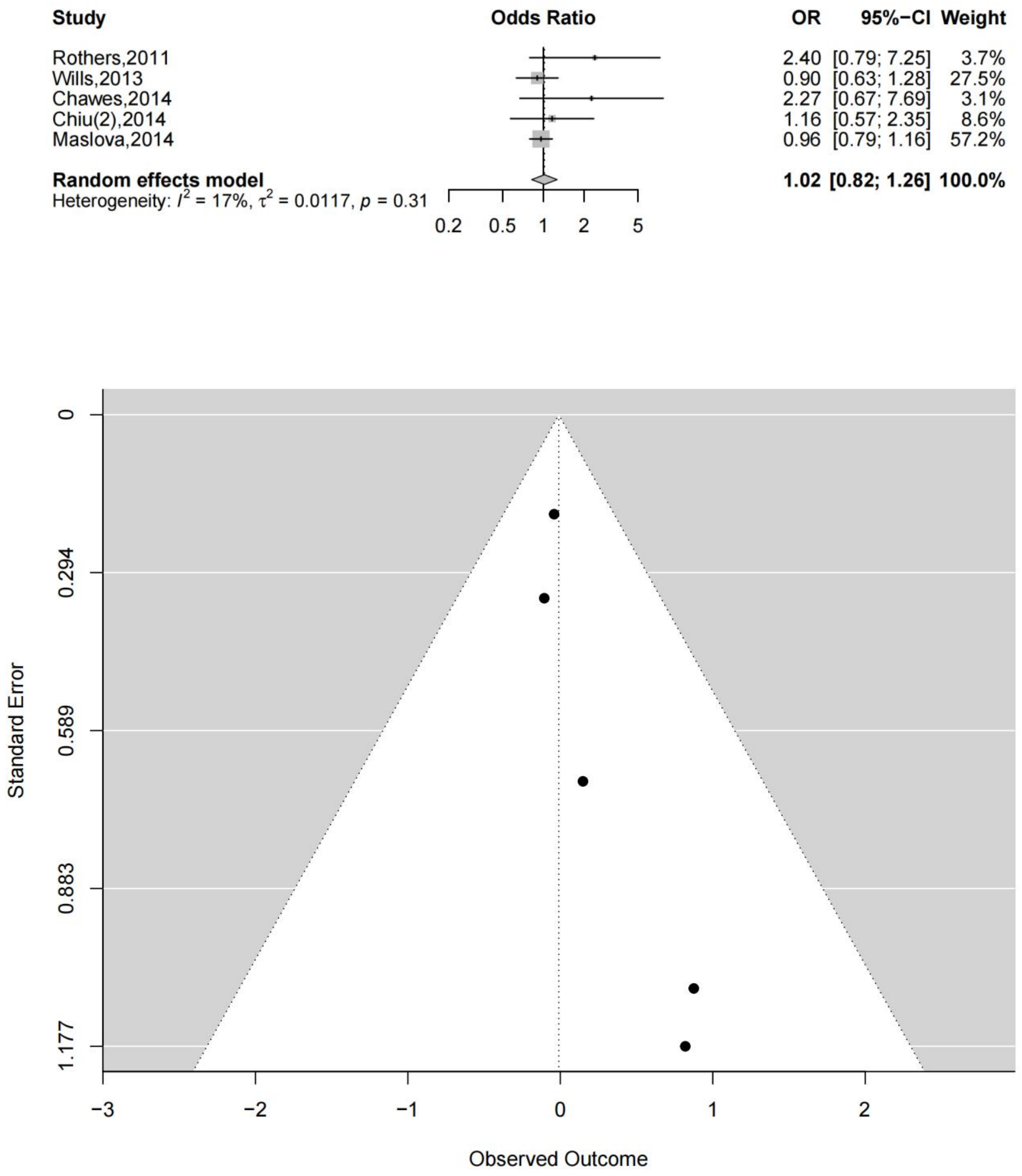

Figure S8. Random-effects meta-analysis of the association between prenatal vitamin D level and offspring atopic dermatitis (forest plot and funnel plot)

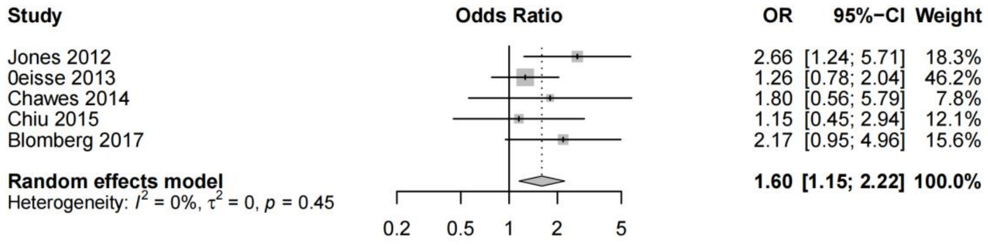

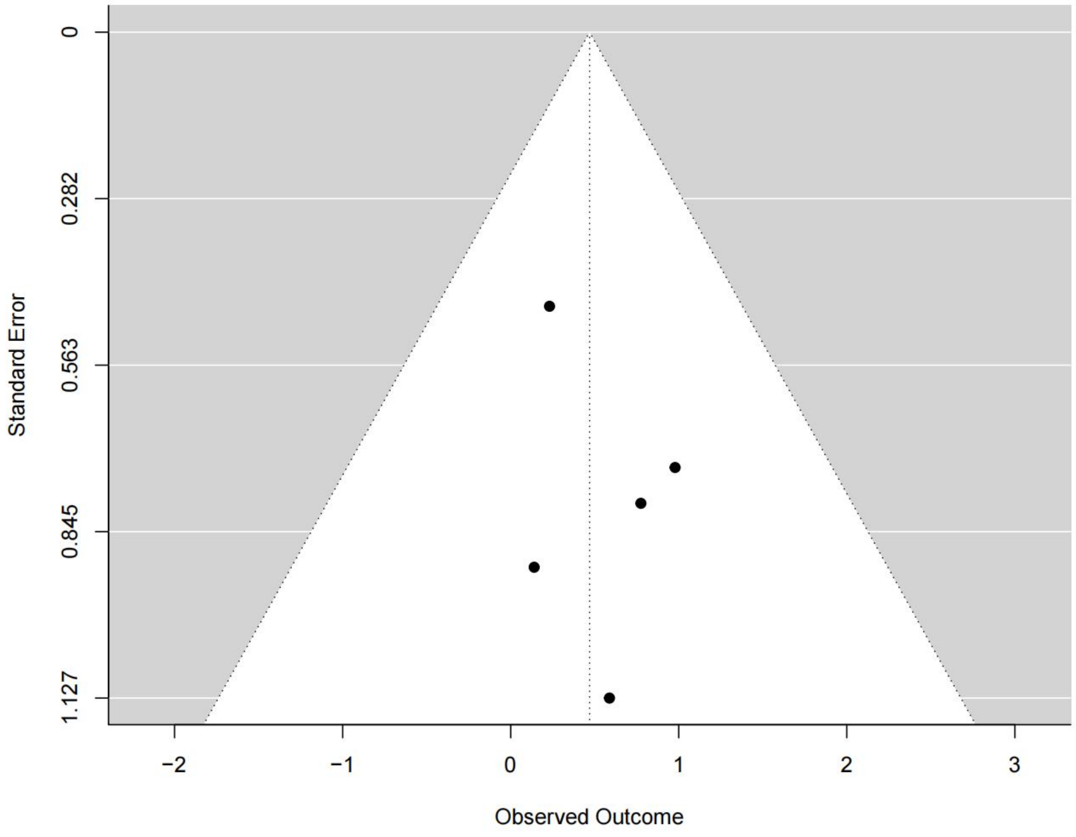

Figure S9. Random-effects meta-analysis of the association between vitamin D supplementation and allergic rhinitis (forest plot and funnel plot)

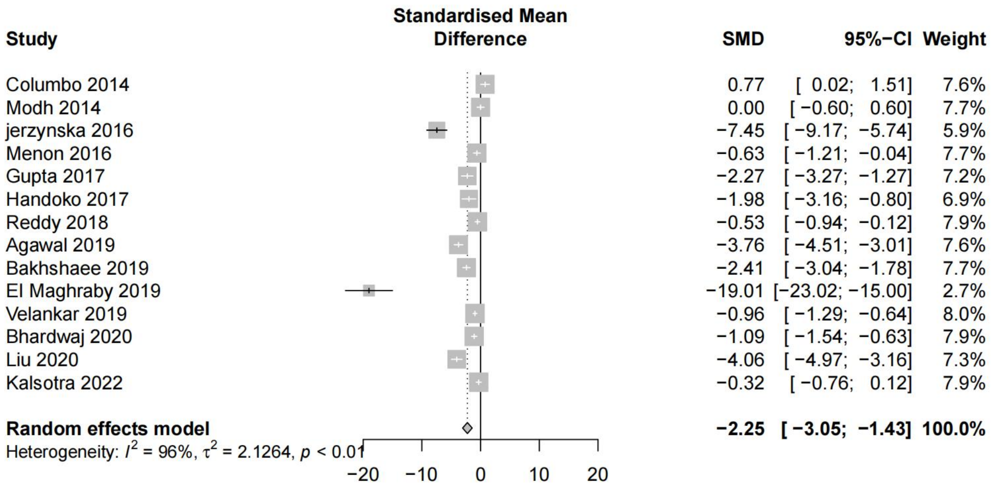

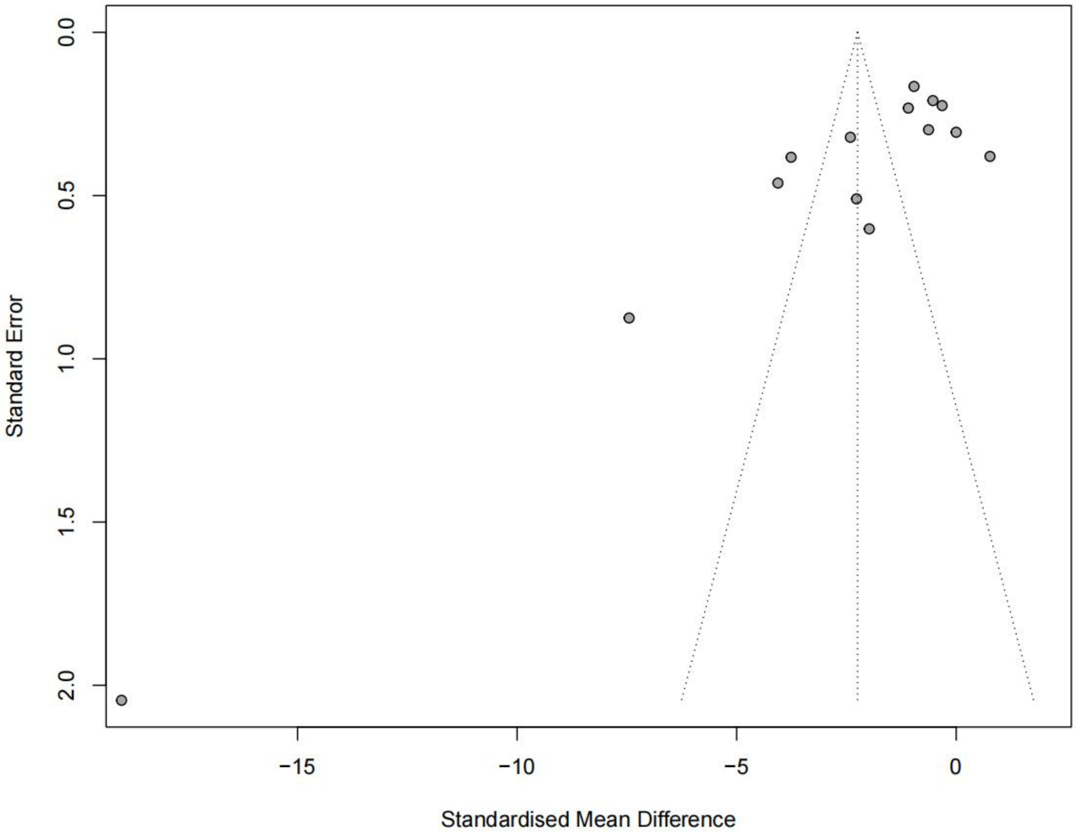

Figure S10. Random-effects meta-analysis of the association between vitamin D supplementation and atopic dermatitis (forest plot and funnel plot)

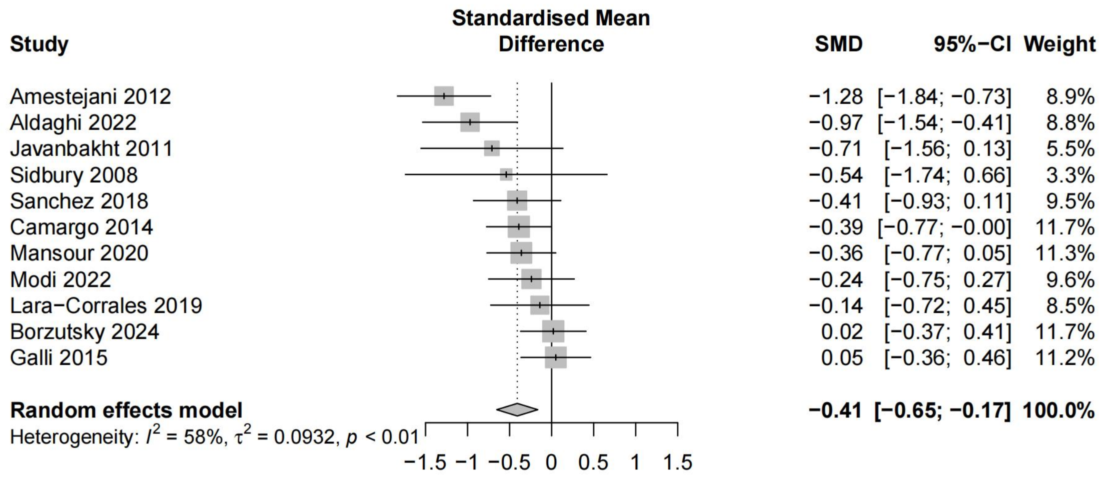

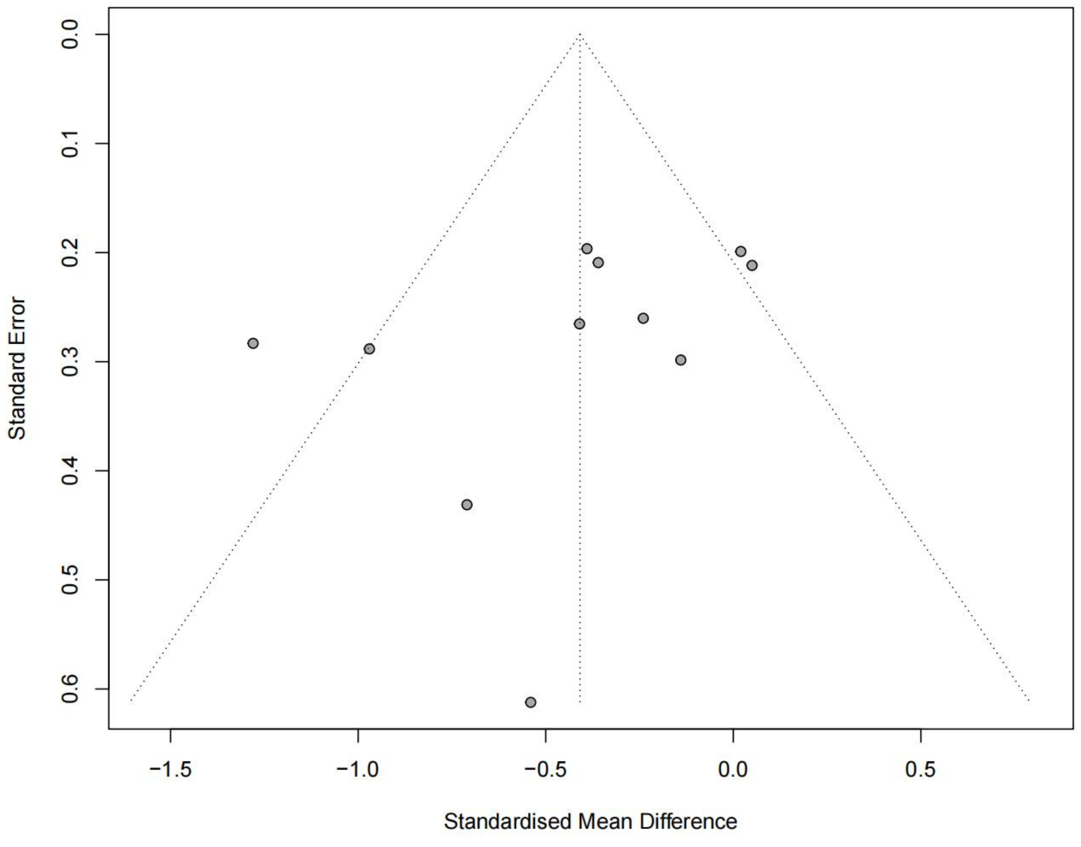

Figure S11. Random-effects meta-analysis of the association between vitamin D supplementation and food allergy (forest plot and funnel plot)

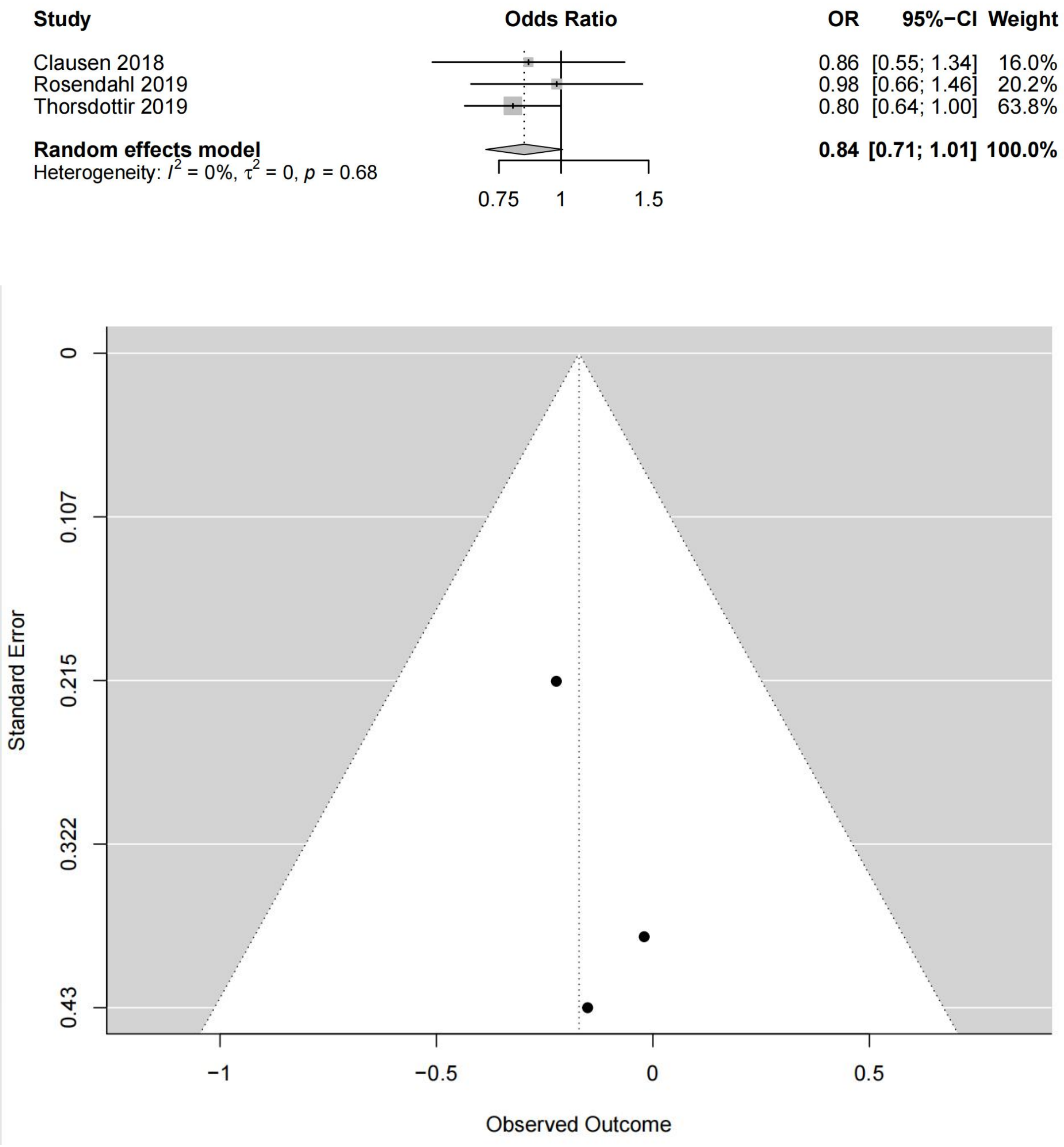

Figure S12. Random-effects meta-analysis of the association between vitamin D supplementation and asthma (forest plot and funnel plot)

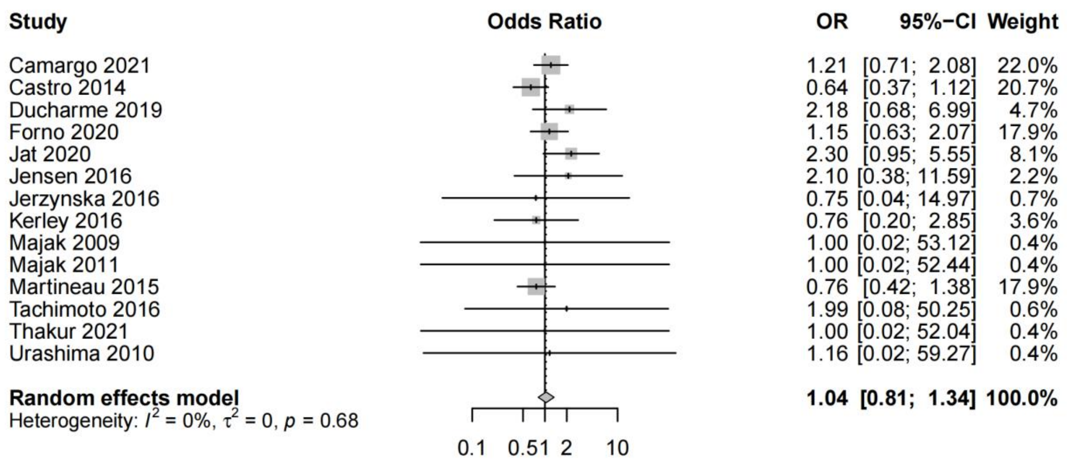

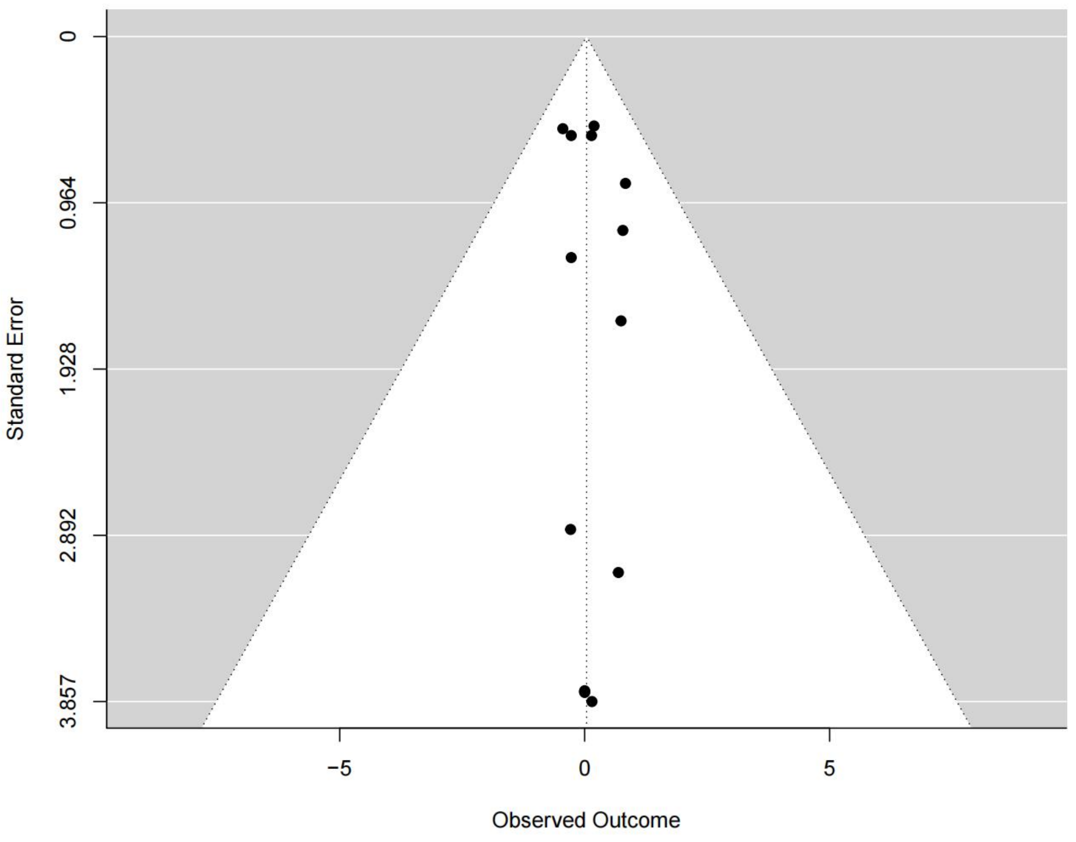

Figure S13. Random-effects meta-analysis of the association between vitamin D supplementation and urticaria (forest plot and funnel plot)

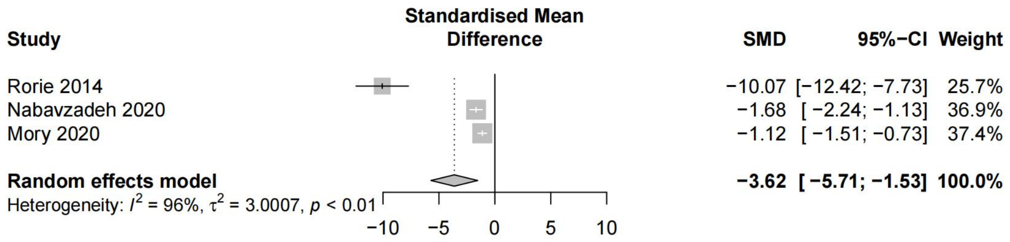

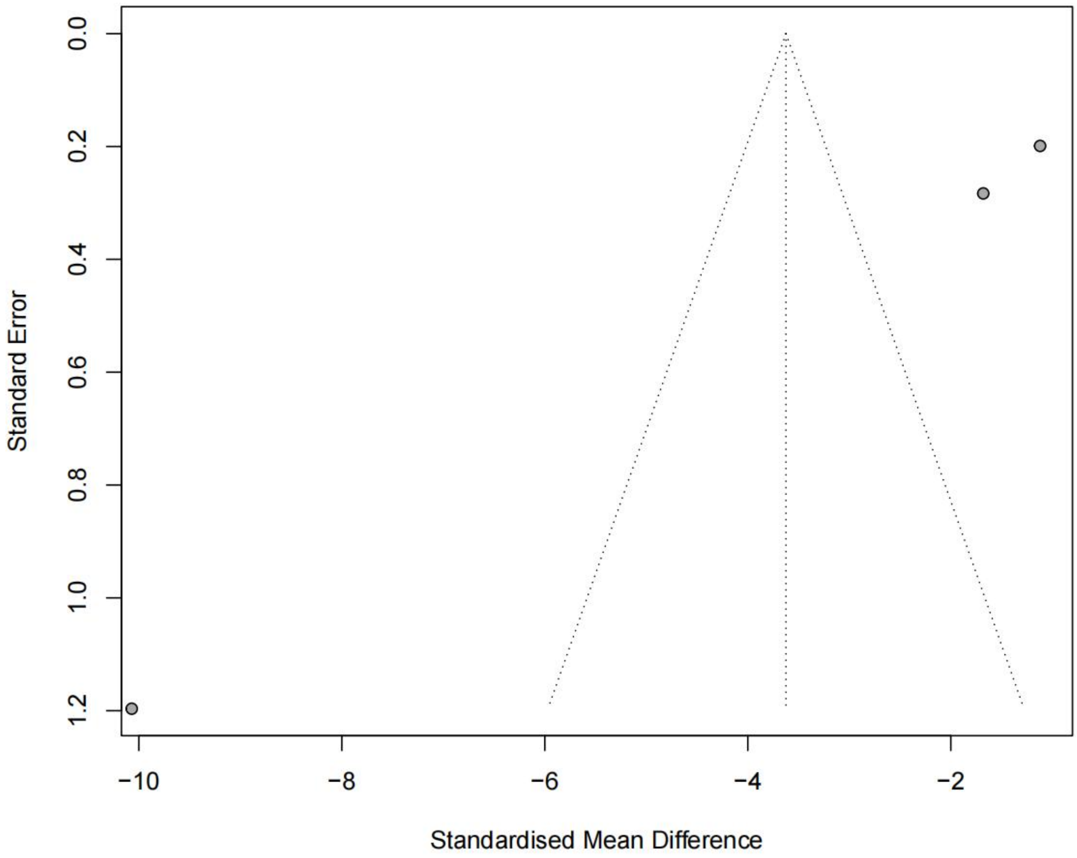

Figure S14. Random-effects meta-analysis of the association between prenatal vitamin D supplementation and allergic rhinitis (forest plot and funnel plot)

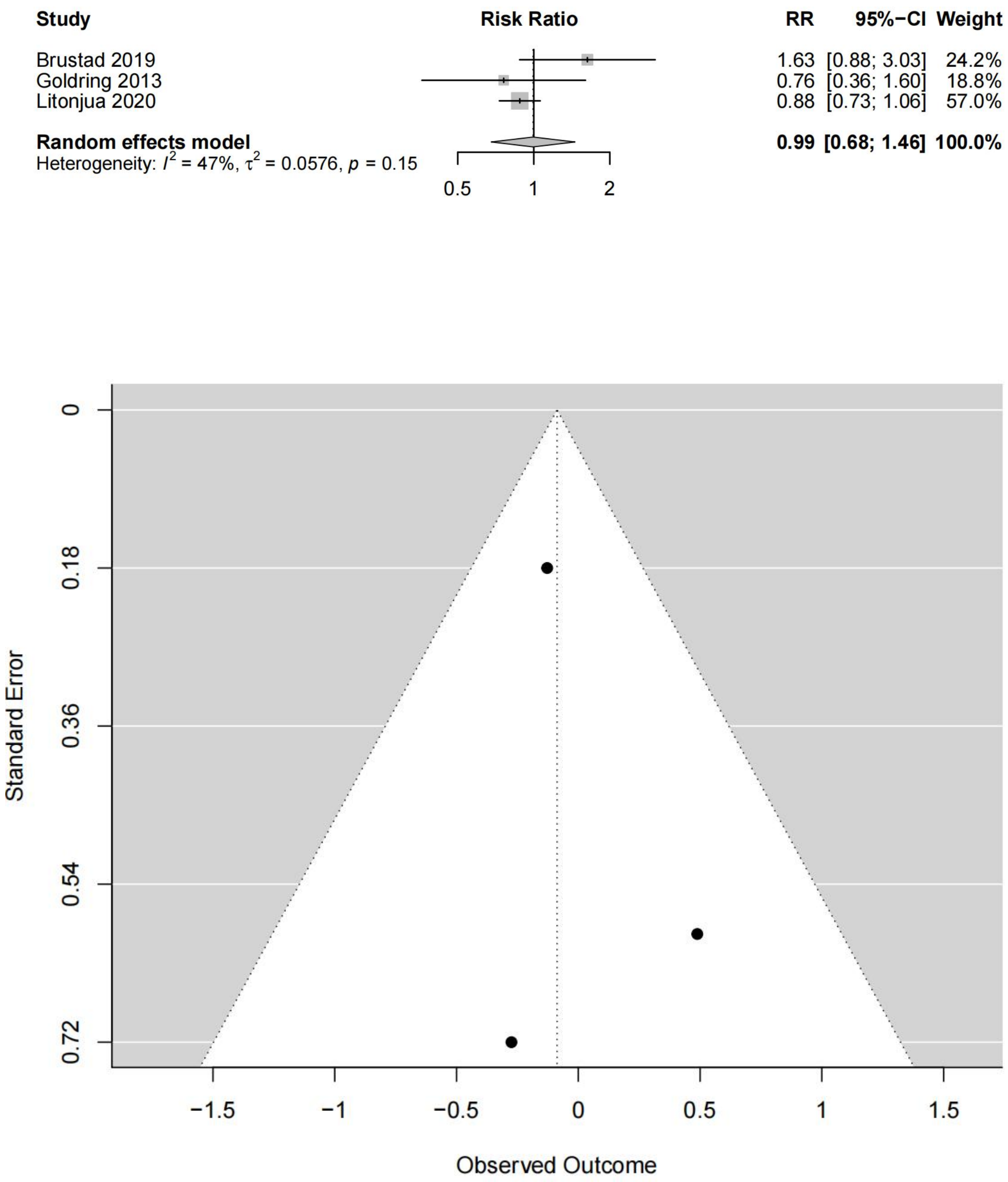

Figure S15. Random-effects meta-analysis of the association between prenatal vitamin D supplementation and atopic dermatitis (forest plot and funnel plot)

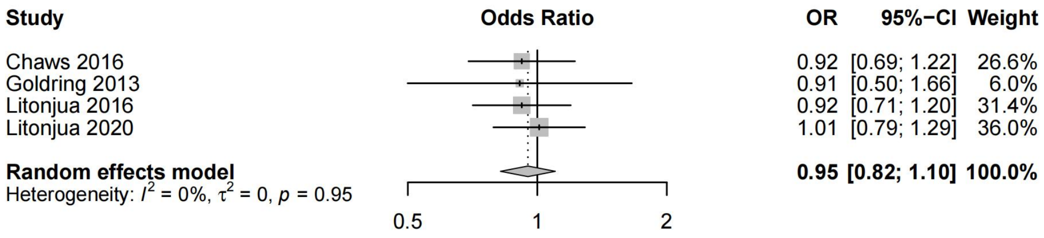

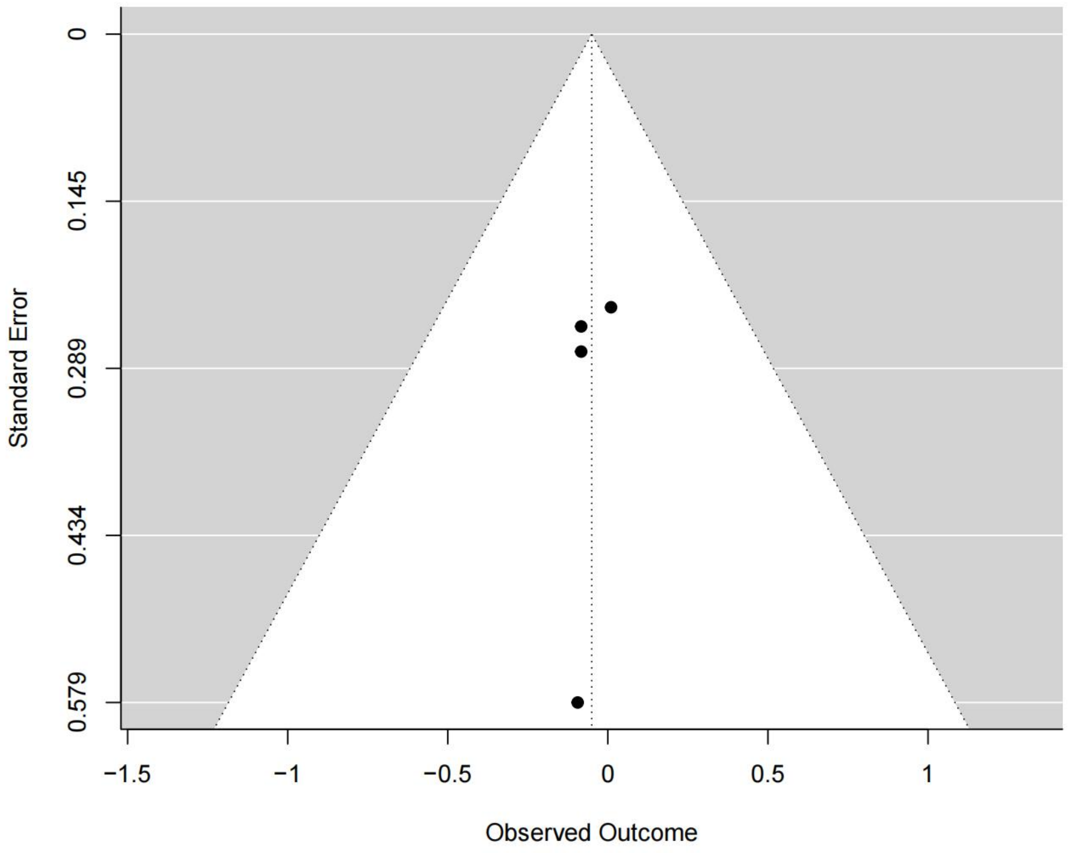

Figure S16. Random-effects meta-analysis of the association between prenatal vitamin D supplementation and asthma (forest plot and funnel plot)

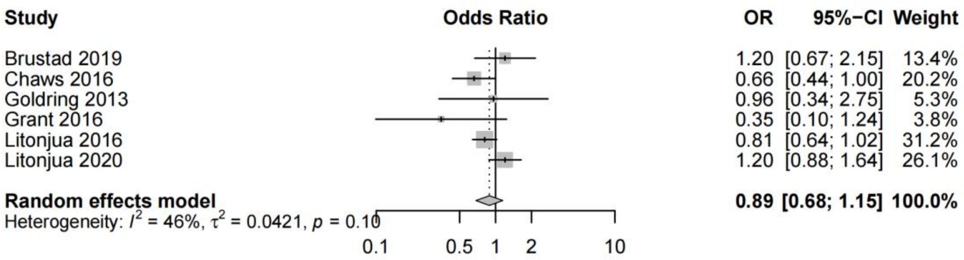

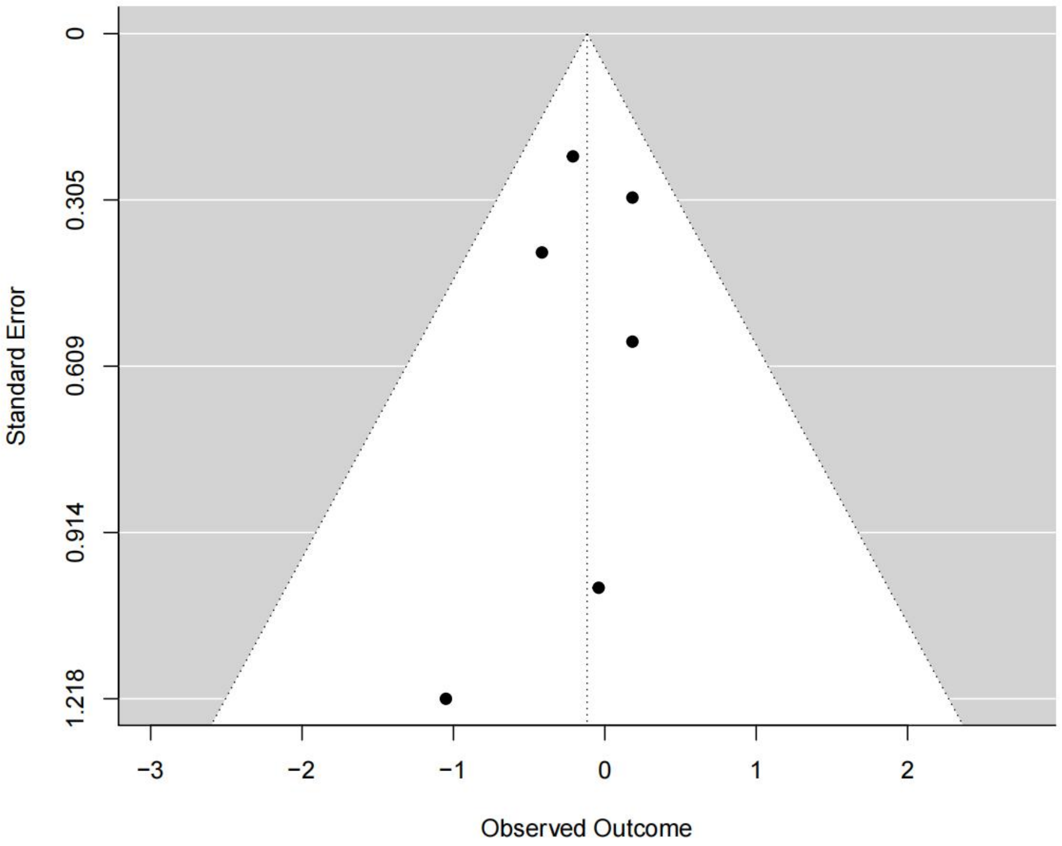

Supplement: Supplementary file 1 [file Datasheet1.pdf]
